# TRIM21 restricts influenza A virus replication by ubiquitination-dependent degradation of M1

**DOI:** 10.1371/journal.ppat.1011472

**Published:** 2023-06-21

**Authors:** Lulu Lin, Xingbo Wang, Zhen Chen, Tingjuan Deng, Yan Yan, Weiren Dong, Yu Huang, Jiyong Zhou

**Affiliations:** 1 MOA Key Laboratory of Animal Virology, Zhejiang University Center for Veterinary Sciences, Hangzhou, China; 2 State Key Laboratory for Diagnosis and Treatment of Infectious Diseases, Zhejiang University First Affiliated Hospital, Hangzhou, China; 3 Institute of Animal Husbandry and Veterinary, Fujian Academy of Agricultural Sciences, Fuzhou, China; Emory University School of Medicine, UNITED STATES

## Abstract

Tripartite motif-containing protein 21 (TRIM21), an E3 ubiquitin ligase, plays a critical role in the host antiviral response. However, the mechanism and antiviral spectrum of TRIM21 in influenza A virus (IAV) remain unclear. Here, we report that TRIM21 inhibits the replication of various IAV subtypes by targeting matrix protein 1 (M1) from H3/H5/H9, but not H1 and H7 M1. Mechanistically, TRIM21 binds to the residue R^95^ of M1 and facilitates K48 ubiquitination of M1 K^242^ for proteasome-dependent degradation, leading to the inhibition of H3, H5, and H9 IAV replication. Interestingly, the recombinant viruses with M1 R^95^K or K^242^R mutations were resistance to TRIM21 and exhibited more robust replication and severe pathogenicity. Moreover, the amino acid sequence M1 proteins, mainly from avian influenza such as H5N1, H7N9, H9N2, ranging from 1918 to 2022, reveals a gradual dominant accumulation of the TRIM21-driven R^95^K mutation when the virus jumps into mammals. Thus, TRIM21 in mammals’ functions as a host restriction factor and drives a host adaptive mutation of influenza A virus.

## Author summary

IAVs use various adaptive mutation strategies to evade host restriction. In the present study, we found that TRIM21 in mammals directly bound to M1 R^95^ site resulted in proteasome-degradation of M1 via K48-linked ubiquitination at K^242^ and thereby inhibited replication of IAV, which caused inhibitory pressure for host adaptive mutation of IAVs at R^95^ of M1. This study highlights the association between host restriction factor and virus evolution.

## Introduction

Influenza A virus (IAV), a respiratory pathogen, belongs to the *Orthomyxoviridae* family and exhibits the characteristics of seasonal transmission, leading to devastating morbidity and mortality worldwide. In recent years, in addition to H5N1, H7N9 and H9N2 avian influenza viruses, some new avian-origin IAV strains, such as H5N6, H5N8 and H10N8 [1–3], have been identified to cause a severe disease due to the high pathogenicity in humans. These avian-origin IAV strains cause a global public health concern with endemic potential.

IAV possesses an eight-segment, negative-sense single stranded RNA genome encoding ten essential proteins and up to eight accessory proteins [4,5]. Matrix 1 (M1) is the most abundant structural protein located inside the envelope membrane in virions [6,7]. M1 has many functions in the process of uncoating, transcription, nuclear export of viral ribonucleoproteins (vRNPs), assembly and budding during the life cycle of influenza virus [7–14], including the formation of the matrix layer during virus assembly and budding processes [13,14], and nuclear-cytoplasmic shuttling that mediates the nuclear export of RNPs [10,11]. The interaction of M1 with vRNPs and the cytoplasmic tail of transmembrane viral protein is related to the formation and release of virus particles [12,15–17]. Additionally, M1 binds to the endoplasmic reticulum and participates in the formation of progeny virus particles [18]. The high conservation of M1 in different IAV subtypes means that it might be a potential broad-spectrum antiviral target.

Tripartite motif (TRIM) proteins are a family of ubiquitin E3 ligases that play critical roles in diverse cellular processes such as innate immunity, cell differentiation, and apoptosis. TRIM proteins are characterized by a tripartite domain structure consisting of a RING domain, a B-box domain, and a coiled-coil domain, followed by a variable C-terminal domain. The C terminal of most TRIMs contains a PRY/SPRY domain. The RING domain confers E3 ligase activity, which enables TRIM proteins to ubiquitinate target proteins, leading to their degradation by proteasome or lysosome, or alteration of their subcellular localization or function [19]. The B box and CC domains are required for oligomerization and the PRY/SPRY domain mediated the interaction between TRIMs and proteins or RNA [20].

The TRIM family of proteins play important roles in the host defense against viral infection. The antiviral strategies of TRIM proteins involve the modulation of innate immunity, direct restriction of viruses, and autophagy-mediated antiviral defense [21]. As E3 ubiquitin ligases, TRIM proteins inhibit viral replication by directly targeting viral proteins. For example, TRIM52 targets the nonstructural protein 2A (NS2A) to restrict Japanese encephalitis virus via proteasomal degradation [22]. TRIM56 is reported to limit the replication of human coronavirus OC43, HIV-1, yellow fever virus, bovine viral diarrhea virus, and dengue virus [21,23–25]. TRIM32 and TRIM35 target polymerase subunits PB1 and PB2 to restrict IAV replication via proteasomal degradation [26,27]. TRIM22 and TRIM41 inhibit IAV infection by targeting the nucleoprotein (NP) for ubiquitin-mediated degradation [28,29]. In addition, TRIM56 restricts IAV infection by blocking IAV RNA synthesis [30]. Nuclear TRIM25 specifically targets influenza virus ribonucleoproteins to block the onset of RNA chain elongation [31]. TRIM21 is reported to be important in the antiviral response against adenoviruses, rhinoviruses, rotaviruses and lymphocytic choriomeningitis virus (LCMV). Thus, on the one hand, the recognition of intracellular antibodies by TRIM21 activates immune signaling. TRIM21 catalyzes the formation of lysine (K) 63-linked polyubiquitination and activates nuclear factor kappa B(NFκB), activator protein 1 (AP1), and interferon regulatory factors (IRF3, IRF5, and IRF7) [32,33]. On the other hand, TRIM21 also targets viral proteins for proteasomal degradation [34,35]. However, the effect of TRIM21 on IAV replication is unclear.

Viruses employ various adaptive mutation strategies to evade host restriction. Pandemic influenza is caused by either reassortment or direct host adaption processes. Host adaptive mutations have been identified in the different stages of influenza virus life cycles [36,37]. The host adaptive mutations of polymerase, NP, and non-structural proteins are related to cross-species transmission. For example, the E^627^K, D^701^N, K^526^R, or G^590^S/Q^591^R in PB2 display a higher polymerase activity in mammalian cells than viruses with polymerases lacking these adaptive mutations [38–40]. Some of these characterized mutations are associated with cross-species transmission caused by H3 and H9 subtype avian influenza viruses [40–42]. A previous study reported that the R^95^K mutation in M1 was associated with airborne transmission in ferrets [43]. Intranuclear replication of influenza virus provides more chances to adapt to the host. Therefore, determining the interaction between viruses and host proteins is crucial to understand host adaptation to influenza virus.

In this study, we demonstrated that TRIM21 binding to the residue R^95^ of M1 facilitates K48 ubiquitination of M1 K^242^ for proteasome-dependent degradation, leading to the inhibition of H3, H5, and H9 IAV replication. Meanwhile, by virus rescue system, we further verified that the R^95^-to-K mutation in the M1 protein was essential for shielding the roles of TRIM21-mediated ubiquitination of M1 K^242^, which benefits viral replication.

## Results

### TRIM21 selectively binds to the M1 protein of different influenza A virus subtypes

**Fig 1:**
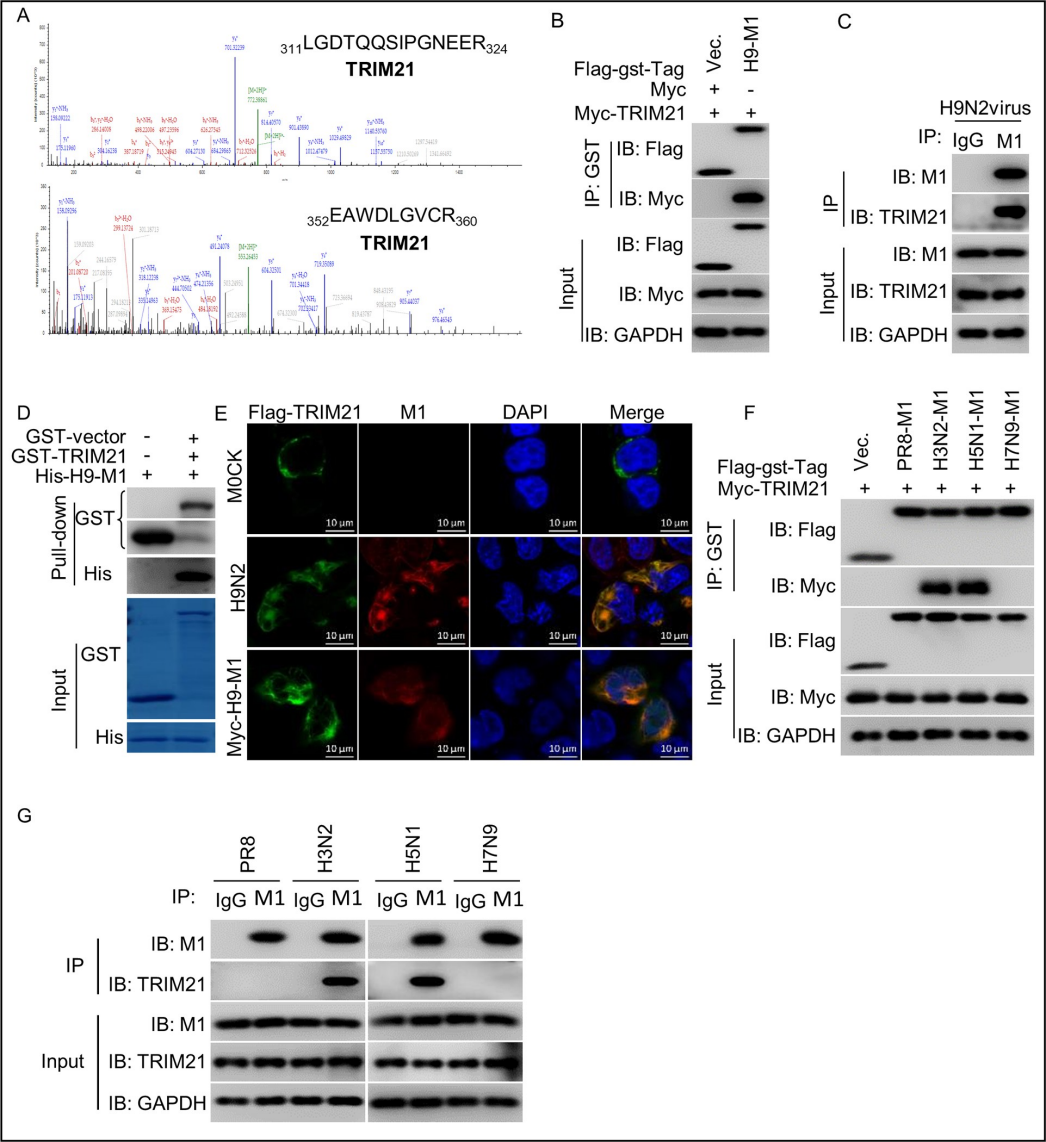
TRIM21 directly interacts with the M1 protein of H9N2, H3N2, and H5N1 viruses. (A) Mass spectrometry identification of TRIM21 as a potential binding partner of H9N2 M1. A549 cells infected with H9N2 virus at an MOI = 1.0 for 24 h were immunoprecipitated with anti-M1 mAb or mouse IgG, and analyzed by mass spectrometry. The TRIM21 was detected in anti-M1 IP samples. Red indicates matched B ions, blue indicates matched Y ions, green indicates unmatched ions, grey indicates precursor ions. (B) TRIM21 interacts with H9N2 M1. Myc-tagged TRIM21, Flag-GST-tagged H9N2 M1 plasmids were transfected into HEK293T cells individually. Cell lysates were subjected to coimmunoprecipitation and western blotting using the indicated antibodies. (C) Endogenous association of TRIM21 with H9N2 M1. A549 cells infected with H9N2 virus at an MOI = 1.0 for 24 h were immunoprecipitated with anti-M1 or control IgG, and analyzed by western blotting with the indicated antibodies. (D) TRIM21 interacts directly with H9N2 M1. Purified His-H9-M1 protein mixed with the GST or GST-TRIM21 proteins was pulled down with GST Sepharose, and analyzed by western blotting with the corresponding antibodies. (E) TRIM21 colocalizes with H9N2 M1. HEK293T cells were transfected with Flag-tagged TRIM21 plasmid for 24 h, infected with H9N2 virus at an MOI = 1.0 for 12 h, or co-tranfected with Flag-tagged TRIM21 and Myc-tagged H9 M1 for 24h, and then incubated with the anti-Flag rabbit mAb, anti-Myc mouse mAb, anti-M1 mouse mAb, FITC-Labeled goat anti-rabbit IgG, and Alexa Fluor 546-conjugated donkey anti-mouse IgG. DAPI staining revealed the nuclei. The images were obtained by confocal microscopy. Scale bar = 10 mm. (F) TRIM21 interacts with H3N2 M1 and H5N1 M1. Myc-tagged TRIM21 and Flag-GST-tagged PR8 M1 or H3N2 M1, H5N1 M1, H7N9 M1 plasmids were individually transfected into HEK293T cells. Cell lysates were subjected to coimmunoprecipitation and western blotting using the indicated antibodies. (G) Endogenous association of TRIM21 with H3N2 M1 and H5N1 M1. A549 cells infected with PR8, H3N2, H5N1 and H7N9 viruses at an MOI = 1.0 for 24 h were immunoprecipitated with anti-M1 or control IgG, and analyzed by western blotting with the indicated antibodies. Each experiment was independently performed with three biological repeats.

It has been reported that the M1 protein of IAV is important for viral replication by participating in the formation of the matrix layer during virus assembly and budding [10,11]. To identify host proteins that bind to M1 of H9N2 IAV, anti-M1 immunoprecipitation (IP) was performed in A549 cells infected with H9N2 virus. Mass spectrometry showed that TRIM21 could be detected in anti-M1 IP samples (Fig 1A). To verify the interaction between M1 and TRIM21, we carried out coimmunoprecipitation (co-IP) assays of the Myc-tagged TRIM21 and Flag-glutathione-S-transferase (GST)-tagged H9N2 M1 (H9-M1) in HEK293T cells. The result showed that TRIM21 bound to H9-M1 (Fig 1B). Similarly, in H9N2 virus-infected cells, a co-IP assay using an anti-M1 monoclonal antibody (mAb) also showed that the M1 protein of H9N2 virus interacted with TRIM21 (Fig 1C). To further determine whether the interaction is direct, purified GST-TRIM21 and His-H9-M1 proteins were subjected to a GST pull down assay. The result showed that TRIM21 directly binds to H9N2 M1 (Fig 1D). Moreover, confocal microscopy revealed colocalization between TRIM21 and M1, when TRIM21 was overexpressed in HEK293T cells infected with H9N2 virus or co-transfected with Flag-TRIM21 and Myc-H9N2-M1 plasmids (Fig 1E). These data demonstrated that TRIM21 has a direct interaction with the M1 protein of H9N2.

However, it is unclear whether TRIM21 specifically binds to the M1 protein of H9N2 virus. To investigate this, we carried out co-IP assay of the Myc-tagged TRIM21 and Flag-GST-tagged PR8 M1 (PR8-M1), or Flag-GST-tagged H3N2 M1(H3-M1), H5N1 M1 (H5-M1), H7N9 M1 (H7-M1) in HEK293T cells. Unexpectedly, in the co-IP assay, TRIM21 was found to bind to the M1 protein from H3N2 virus and H5N1 virus, but not PR8 virus and H7N9 virus (Fig 1F). Similar results were obtained in H3N2- and H5N1-infected cells, but not PR8- and H7N9-infected cells (Fig 1G), indicating that TRIM21 selectively binds to the M1 protein of H3N2 and H5N1 viruses rather than those of PR8 and H7N9 viruses. Together, these data demonstrated that TRIM21 selectively attaches to the M1 proteins of H3N2, H5N1, and H9N2 viruses rather than PR8 and H7N9 viruses.

### The M1 R^95^ residue is necessary for interaction with TRIM21

**Fig 2:**
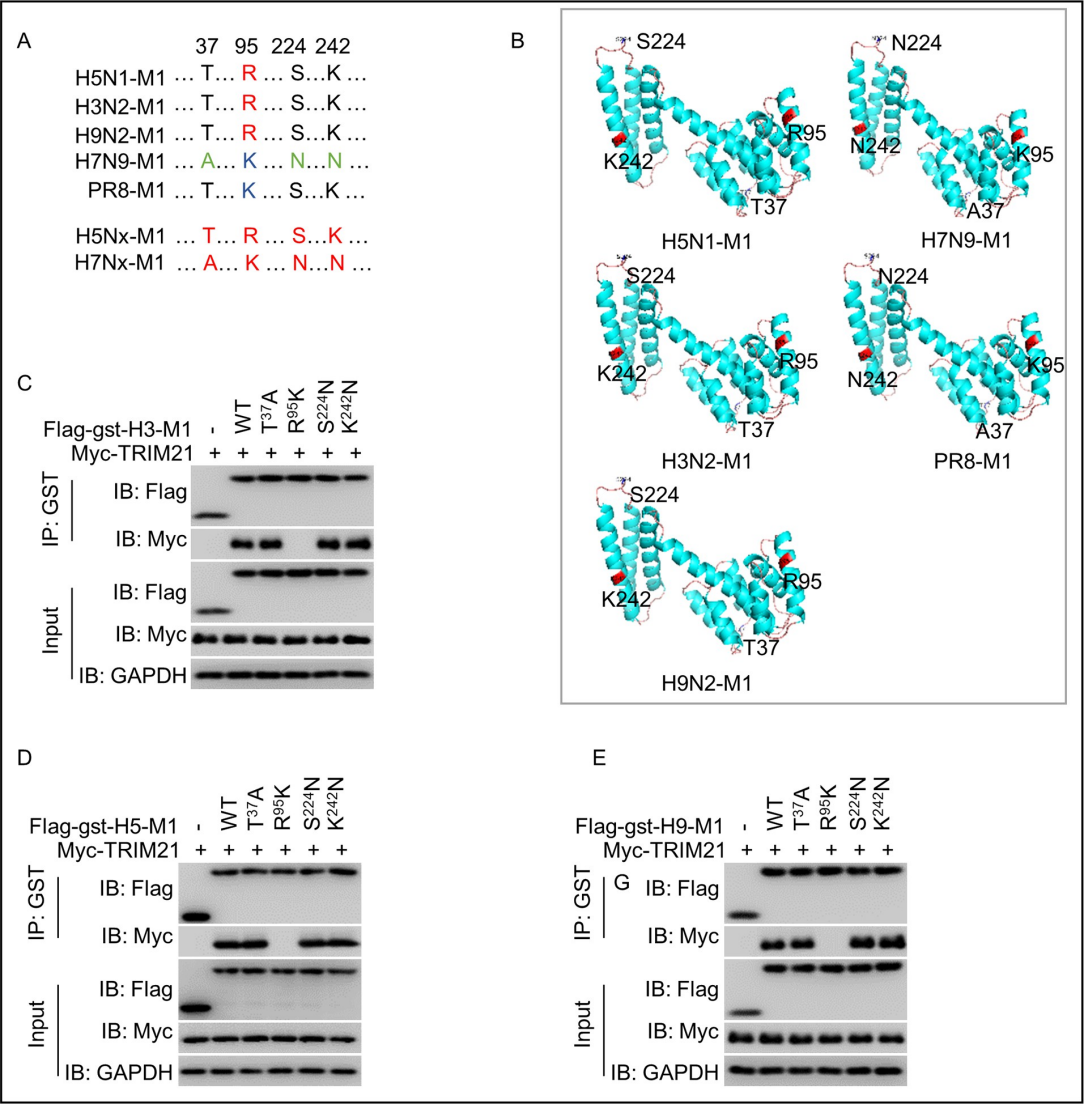
R^95^ of M1 protein is critical for interacting with the PRY/SPRY domain of TRIM21. (A) Amino acid sequence alignment of the M1 protein from various IAV, including PR8, H3N2, H5N1, H7N9, H9N2, H5Nx, and H7Nx. (B) The structural prediction of the M1 proteins from PR8, H3N2, H5N1, H7N9, and H9N2 viruses using SWISS MODEL and PyMOL software. (C-E) R^95^ of M1 is required for the TRIM21 interaction. HEK293T cells were separately transfected with plasmids encoding Flag-GST H3 M1, H5 M1, H9 M1 and its mutants (T^37^A, R^95^K, S^242^N, K^242^N), Myc-tagged TRIM21 plasmid for 48 h individually, and then cell lysates were incubated as indicated for a coimmunoprecipitation assay and analyzed by western blotting with the indicated antibodies. Each experiment was independently performed with three biological repeats.

To investigate why the M1 protein of H3N2, H5N1 and H9N2 viruses rather than PR8 and H7N9 viruses interact with TRIM21, we analyzed the amino acid sequence of M1 proteins from different subtypes of IAV. In the sequence alignment, the residue 95 is lysine (K) in the M1 protein of PR8 and H7 subtypes, but arginine (R) in the M1 protein of H3, H5 and H9 subtypes. Additionally, the residues ^37^alanine (A), ^224^asparagine (N) and ^242^N in the M1 protein of H7N9 are distinct from the ^37^threonine (T), ^224^serine (S) and ^242^K in PR8, H3N2, H5N1, and H9N2 IAV subtypes (Fig 2A). Interestingly, we observed that the residues T^37^, R^95^, S^224^ and K^242^ in the M1 protein of H5Nx were distinct from the residues A^37^, K^95^, N^224^, and N^242^ in the M1 protein of H7Nx (Fig 2A). We further simulated the three-dimensional structure of the M1 proteins from different subtypes of IAV using PyMOL software. However, the three-dimensional structure of the M1 protein of H3N2, H5N1 and H9N2 viruses did not differ from those of PR8 and H7N9 viruses (Fig 2B).

To finely detect whether these four aforementioned amino acid residues in the M1 protein are involved in binding to TRIM21, we constructed the M1 mutants T^37^A, R^95^K, S^224^N, and K^242^N of H3N2, H5N1, and H9N2 viruses by replacing the corresponding residues of H3N2, H5N1, and H9N2 M1 proteins with those of H7N9 virus. We generated the vectors expressing the Flag-GST-M1 mutants T^37^A, R^95^K, S^224^N, and K^242^N individually. Plasmids encoding these M1 mutants and Myc-tagged TRIM21 were transfected into HEK293T cells. Co-IP assay showed that the mutants T^37^A, S^224^N, and K^242^N, but not mutant R^95^K, interacted with TRIM21 (Figs 2C–2E). These data indicated that the residue R^95^ of M1 in H3N2, H5N1, and H9N2 is a crucial binding site for TRIM21.

### The M1 R^95^ residue is required for TRIM21-mediated K48-linked polyubiquitination-dependent degradation of M1

**Fig 3:**
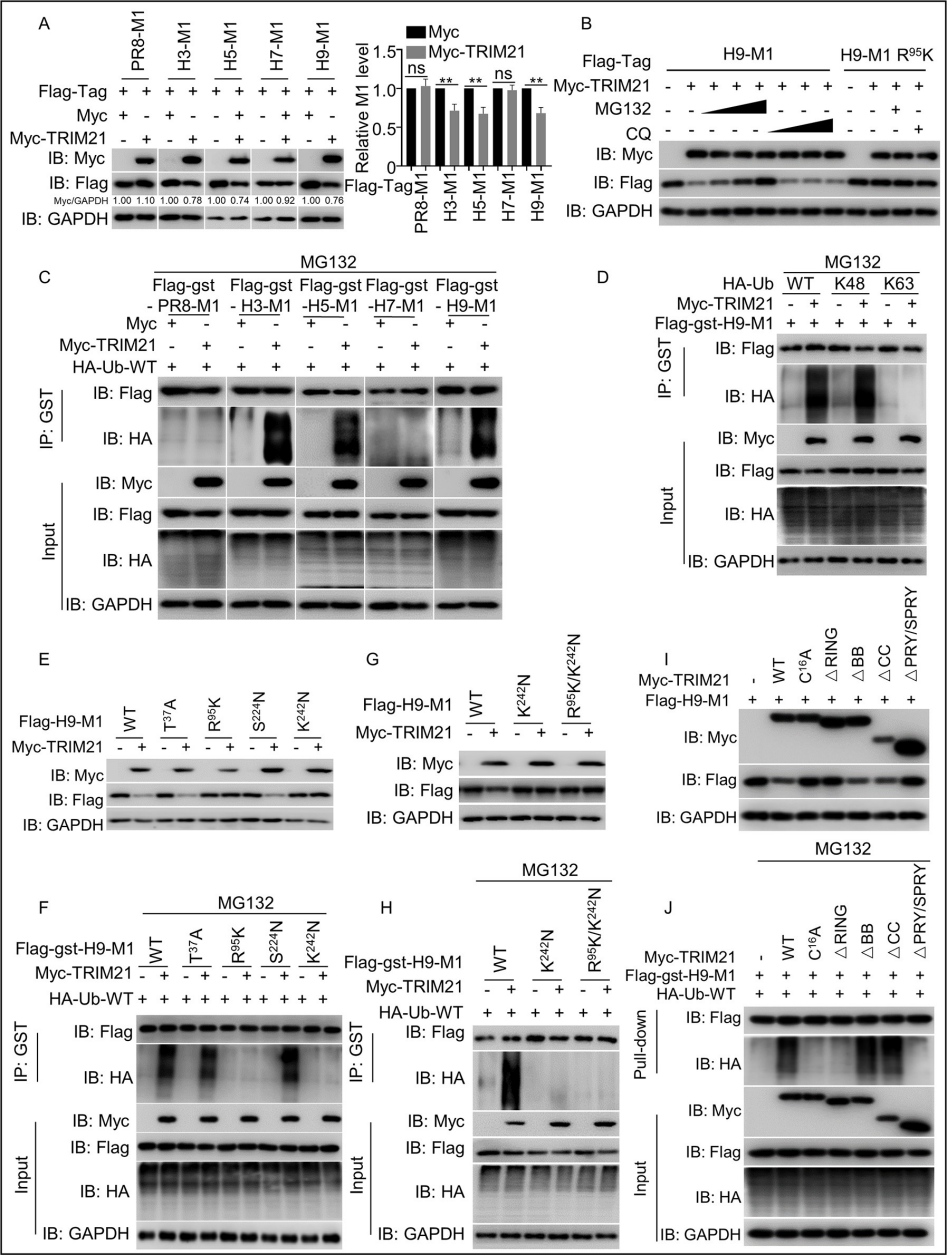
TRIM21 ubiquitinates H9N2 M1 via the R^95^ and K^242^ residues. (A) TRIM21 degrades H3/H5/H9 M1 rather than H1/H7 M1. Myc-tagged vector or Myc-tagged TRIM21 and Flag-tagged H1/H3/H5/H7/H9 M1 were co-transfected into HEK293T cells, then the cell lysates were detected using the indicated antibodies. The number under the gel band represent the relative protein concentration calculated using Image J software with the gray value of GAPDH as the control standard. (B) TRIM21 mediates H9N2 M1 degradation through the proteasomal pathway. HEK293T cells expressing Flag-tagged H9N2 M1 were transfected with Myc-tagged vector or Myc-tagged TRIM21 for 12 h, followed by treatment with MG132 (6.25μM, 12.5μM, 25μM) or chloroquine phosphate (12.5μM, 25μM, 50μM) for 6 h. Additionally, HEK293T cells expressing Flag-tagged H9N2 M1 R^95^K were co-transfected with Myc-tagged vector or Myc-tagged TRIM21 for 12 h, followed by treatment with MG132 (25μM) or chloroquine phosphate (50μM) for 6 h. The cell lysates were detected using the indicated antibodies. (C) TRIM21 ubiquitinates the M1 protein of H3N2, H5N1 and H9N2 viruses, but not those of H7N9 and PR8 viruses. Myc-tagged vector or the TRIM21 vector was co-transfected with Flag-GST-tagged H9N2 M1 or H7N9 M1, H5N1 M1, H3N2 M1, PR8 M1 and HA-tagged Ub-WT plasmids into HEK293T cells for 48 h, followed by treatment with 25μM MG132 for 6 h. The cell lysates were then subjected to immunoprecipitation and western blotting using the indicated antibodies. (D) TRIM21 promotes K48-linked polyubiquitination of H9N2 M1. Myc-tagged vector or the TRIM21 vector was co-transfected with Flag-GST-tagged H9N2 M1 and HA-tagged Ub-WT or Ub-K48 or Ub-K63 plasmids into HEK293T cells for 48 h, followed by treatment with 25μM MG132 for 6 h. The cell lysates were then subjected to immunoprecipitation. (E-H) R^95^ and K^242^ in H9N2 M1 were necessary for TRIM21 degradation. Myc-tagged vector or Myc-tagged TRIM21 and Flag-tagged H9N2 M1 WT or its mutants (T^37^A, R^95^K, S^242^N, K^242^N, R^95^K/K^242^N) were co-transfected into HEK293T cells, and the cell lysates were detected using the indicated antibodies (E and G). R^95^ and K^242^ in H9N2 M1 were required for TRIM21 ubiquitination. Myc-tagged vector and Myc-tagged TRIM21 WT plasmids were co-transfected with Flag-GST-tagged H9N2 M1 WT or its mutants (T^37^A, R^95^K, S^242^N, K^242^N, R^95^K/K^242^N) and HA-tagged Ub-WT plasmids in HEK293T cells for 48 h, followed by treatment with 25μM MG132 for 6 h. The cell lysates were then subjected to immunoprecipitation and western blotting using the indicated antibodies (F and H). (I) E3 ligase activity is indispensable for TRIM21-mediated degradation of H9N2 M1. Myc-tagged vector, Myc-tagged TRIM21 WT, its mutant C^16^A and TRIM21 deletion (ΔRING, ΔBB, ΔCC and ΔPRY/SPRY) and Flag-tagged H9N2 M1 were co-transfected into HEK293T cells for 12h. Then, the cell lysates were identified using the indicated antibodies. (J) E3 ligase activity was required to catalyze the ubiquitination of H9N2 M1. Myc-tagged vector, Myc-tagged TRIM21 WT, its mutant C^16^A and TRIM21 deletion (ΔRING, ΔBB, ΔCC and ΔPRY/SPRY) were co-transfected with Flag-gst-tagged H9N2 M1 and HA-tagged Ub-WT in HEK293T cells for 48 h, followed by treatment with 25μM MG132 for 6 h. Subsequently, the cell lysates were subjected to immunoprecipitation and western blotting using the indicated antibodies. Each experiment was independently performed with three biological repeats. All results are presented as means ± SD. *, *p <* 0.05; **, *p <* 0.01; ns, *p >* 0.05.

Given that the TRIM21, an E3 ligase in ubiquitination system, interacts with the R^95^ residue of M1, we inferred that TRIM21 regulated degradation of M1 by the ubiquitination system. Indeed, the immunoblotting assay showed that TRIM21 overexpression promotes degradation of H3N2, H5N1 and H9N2 M1, but not PR8 and H7N9 M1 (Fig 3A). Treatment with MG132 (a proteasome inhibitor), but not chloroquine phosphate (an endosome/lysosome pathway inhibitor), inhibited the decrease in H9N2 M1 amount in a dose-dependent manner in the presence of TRIM21, while it did not affect the amount of M1 R^95^K (Fig 3B). To examine the effects of TRIM21 on M1 ubiquitination, HEK293T cells were co-transfected with plasmids Myc-TRIM21 and HA-tagged the wild-type Ub (Ub-WT) in combination with Flag-GST-H9N2 M1 or PR8 M1, H3N2 M1, H5N1 M1, and H7N9 M1. As shown in Fig 3C, the ubiquitination of H3N2 M1, H5N1 M1, and H9N2 M1, but not PR8 M1 and H7N9 M1, was markedly promoted in TRIM21 overexpressing cells. These data suggested that TRIM21 facilitates the ubiquitination-dependent degradation of M1.

To further determine the type of ubiquitination facilitated by TRIM21 on H9N2 M1, we transfected HEK293T cells with plasmids expressing Myc-tagged TRIM21, Flag-GST-tagged H9N2 M1 and HA-tagged Ub-WT or HA-tagged Ub-K48 or HA-tagged Ub-K63. The data presented in Fig 3D revealed that TRIM21 promotes K48-linked rather than K63-linked ubiquitination of the H9N2 M1. However, in the ubiquitination assay, we observed that TRIM21-mediated ubiquitination degradation disappeared in the H9N2 M1 mutants R^95^K or K^242^N, respectively, but not in the mutants T^37^A and S^224^N (Fig 3E and 3F), demonstrating that ubiquitination of M1 did not occur when TRIM21 did not recognize the amino acid residue K^95^ of the M1 protein. To finely identify the ubiquitin site, we constructed the H9N2 M1 mutants R^95^K/K^242^N. We confirmed that the ubiquitination and degradation of M1 mutants with K^242^N or R^95^K/K^242^N were abolished in comparison with WT H9N2 M1 (Fig 3G and 3H), demonstrating that residue K^242^ is a ubiquitination site of M1 protein mediated by TRIM21, and TRIM21 attachment to R^95^ of the M1 protein is critical for the ubiquitination of M1 at the residue K^242^. In addition, we constructed single arginine substitution mutants at all thirteen lysine sites of H9N2 M1, and observed that all mutants except K^242^R were degraded by TRIM21 (S1A Fig). Co-IP assay confirmed the TRIM21-mediated ubiquitination of WT H9N2 M1 and single amino acid mutants of H9N2 M1 with K^21^R, K^35^R, K^47^R, K^57^R, K^98^R, K^101^R, K^102^R, K^104^R, K^113^R, K^187^R, K^230^R, and K^252^R, but not the H9N2 M1 mutant with K^242^R (S1B Fig). Moreover, the results showed that the ΔBB (Δ92–123 aa) and ΔCC (Δ125–235 aa) of TRIM21 could catalyze the ubiquitination degradation of H9N2 M1, while ΔPRY/SPRY (Δ268–465 aa), a single-amino-acid mutant C^16^A and ΔRING (Δ16–54 aa) of TRIM21 that lost E3 ligase activity could not catalyze the ubiquitination degradation of H9N2 M1 (Fig 3I and 3J), confirming that TRIM21 acted as an E3 ligase in ubiquitination of H9N2 M1. Taken together, R^95^ of M1 is the critical residue for TRIM21 RING domain mediated K48-linked ubiquitination of residue K^242^ of M1.

### The M1 R^95^ residue is essential for TRIM21-mediated restriction of virus replication

**Fig 4:**
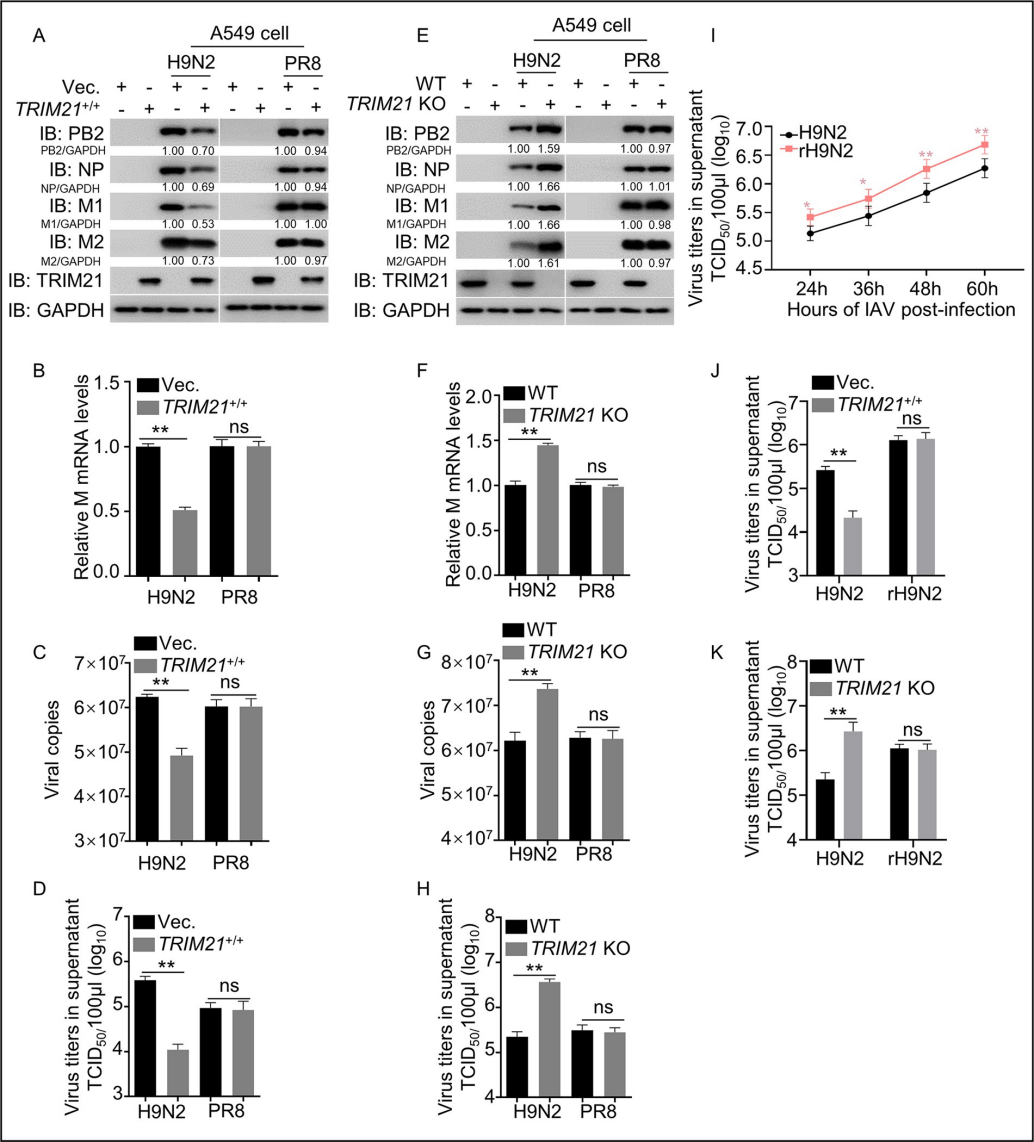
M1 is critical for TRIM21 inhibition of H9N2 virus replication. (A-D) TRIM21 inhibits H9N2 rather than PR8 IAV replication in *TRIM21* overexpressing A549 cells. The *TRIM21* overexpressing A549 cell lines and control cells were infected with H9N2 virus or PR8 virus at an MOI = 1.0 for 12 h and then the levels of protein (A), mRNA (B), vRNA (C) and the TCID_50_ (D) were examined. The number under the gel band is the relative protein concentration calculated using Image J software with the gray value of GAPDH as the control standard. (E-H) TRIM21 increased H9N2 virus replication in *TRIM21*-KO A549 cells. *TRIM21*-KO A549 cells and control cells were infected with H9N2 virus or PR8 virus at an MOI = 1.0 for 12 h, and the levels of protein (E), mRNA (F), vRNA (G), and the TCID_50_ (H) were examined. (I) One-step growth curves of H9N2 and mutant H9N2 (rH9N2). H9N2 and rH9N2 viruses were rescued in HEK293T cells, which were subsequently inoculated and propagated in 9-day-old SPF embryonated chicken eggs. Then, A549 cells were infected with an equal amount of allotonic fluid containing WT H9N2 or rH9N2 at 0.01 MOI for 24, 36, 48, and 60 h, respectively. The cell supernatant was harvested at the indicated time points, and the TCID_50_ was calculated as described in the Materials and Methods. (J-K) TRIM21 restricted H9N2 replication but not rH9N2 in *TRIM21* overexpressing A549 cell lines. *TRIM21* overexpressing, *TRIM21*-KO, and control A549 cells were infected with H9N2 or rH9N2 at an MOI = 1.0 for 12 h and then the viral titers were detected using TCID_50._ *, *p <* 0.05; **, *p <* 0.01; ns, *p >* 0.05. Each experiment was independently performed with three biological repeats. All results are presented as means ± SD.

Given that the TRIM21 mediated proteasome degradation of M1, we investigated whether TRIM21 regulates IAV infection. TRIM21 overexpressing A549 cells were infected with PR8, H3N2, H5N1, H7N9 and H9N2 viruses (MOI = 1.0) for 12 h. Western blotting showed that TRIM21 overexpression decreased the level of viral proteins in H3N2, H5N1, and H9N2-infected A549 cells, but not in PR8 and H7N9-infected cells (Figs 4A and S2A). Consistently, quantitative real-time reverse transcription PCR (qRT-PCR) and 50% tissue infective dose (TCID_50_) assays also showed that TRIM21 overexpression decreased the level of *M1* mRNA, vRNA, and viral titer of H3N2, H5N1, and H9N2, but not PR8 and H7N9 (Figs 4B–4D and S2B–S2D). The similar results were observed in TRIM21 overexpressed HEK293T cells (S3A–S3D Fig). To further confirm the effect of TRIM21 on IAV replication, we generated *TRIM21*-knocked out (*TRIM21*-KO) A549 and HEK293T cell lines. The level of viral proteins, *M1* mRNA, vRNA, and viral titer for H3N2, H5N1 and H9N2 virus but not PR8 and H7N9, were enhanced in *TRIM21* knocked out A549 and HEK293T cell lines, as detected using western blotting, qRT-PCR, and TCID_50_ assays (Figs 4E–4H, S2E–S2H and S3E–S3H). Overall, these data demonstrated that TRIM21 inhibits the replication of H3N2, H5N1 and H9N2 viruses.

To further validate the role of the *M1* gene in TRIM21 inhibition of IAV replication, we replaced the *M1* gene of the H9N2 virus with that of the PR8 virus (named rH9N2). Wild-type (WT) H9N2 (H9N2) and rH9N2 viruses were rescued in HEK293T cells, which were subsequently inoculated and propagated in 9-day-old specific pathogen free (SPF) embryonated chicken eggs. Then, we infected A549 cells with an equal amount of rescued wild-type and mutant viruses at the initial step, collected supernatants at various time points, and used TCID_50_ detection to draw virus replication growth curves. Virus titer detection in A549 cells showed that the rH9N2 replicated more robustly than H9N2, indicating that the *M1* gene of PR8 virus remarkably improved the replication ability of H9N2 (Fig 4I). Subsequently, we tested the replication of rH9N2 virus in *TRIM21*-overexpressing or *TRIM21*-KO A549 cell lines. *TRIM21*-overexpressed or KO A549 cells were infected with the H9N2 and rH9N2 at MOI = 1.0. The results showed that the viral titer of rH9N2 virus was not significantly different in *TRIM21*-overexpressing or KO cells (Fig 4J–4K), indicating that TRIM21 captured the M1 protein to inhibit replication of H9N2, but not rH9N2. Taken together, TRIM21 targeting M1 is critical for TRIM21-mediated inhibition of virus infection.

**Fig 5:**
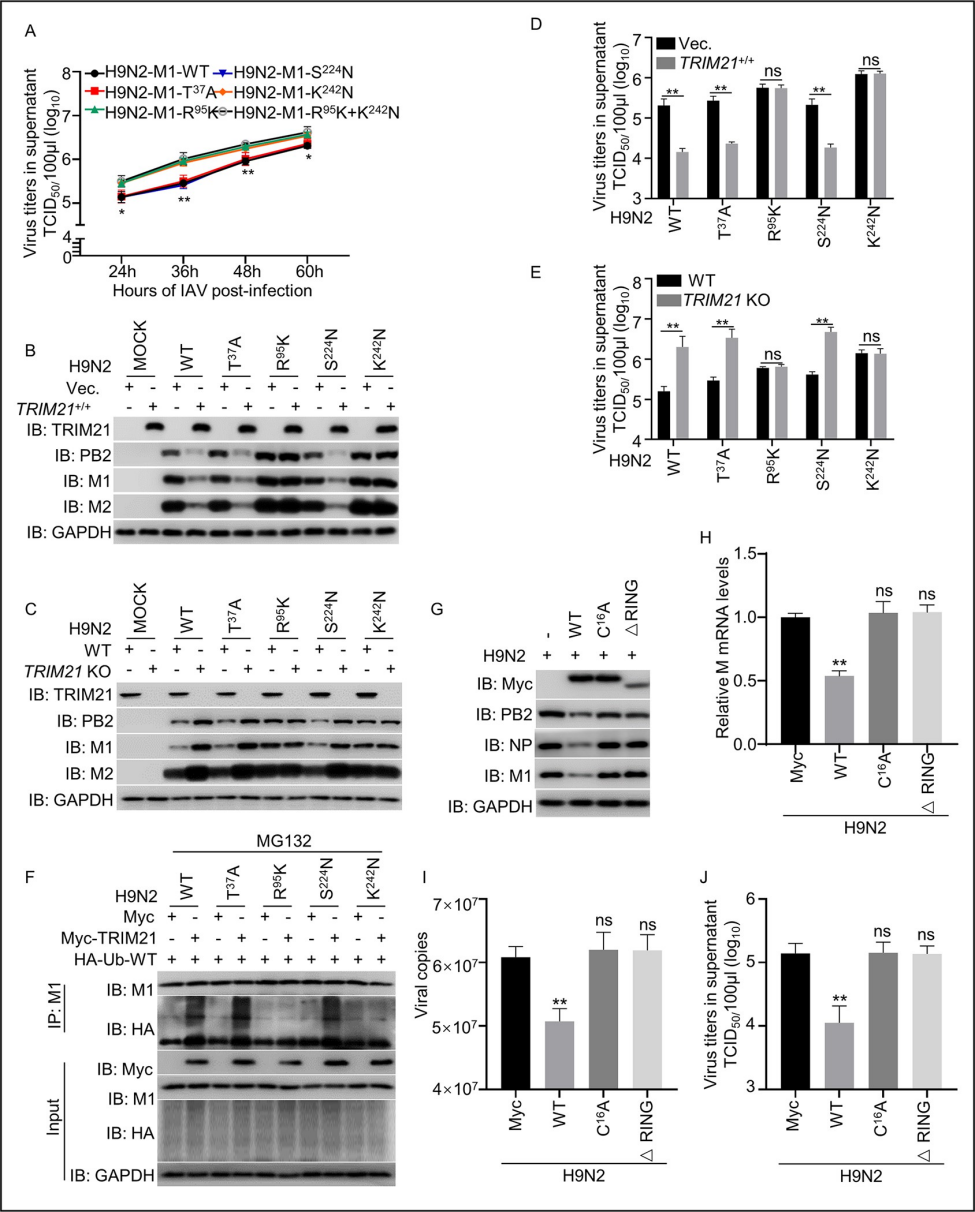
R^95^K^242^ of the M1 protein and the TRIM21 are critical for inhibiting IAV replication by ubiquitination degradation. (A) One-step growth curve of WT and mutant H9N2 viruses. The WT and recombinant viruses were rescued in HEK293T cells and propagated in 9-day-old SPF embryonated chicken eggs. Then, A549 cells were infected with an equal amount of allotonic fluid containing WT and four mutant H9N2 viruses at 0.01 MOI for 24, 36, 48, and 60 h, respectively. The cell supernatant was harvested, and the TCID_50_ was calculated as described in the Materials and Methods. (B-E) TRIM21 restricted the replication of WT and all mutant H9N2 viruses, except R^95^K virus and K^242^N virus, in A549 cells. *TRIM21*-overexpressing, *TRIM21*-KO, and control A549 cells were infected with H9N2 WT and mutants H9N2 M1-T^37^A, H9N2 M1-R^95^K, H9N2 M1-S^224^N, and H9N2 M1-K^242^N at an MOI = 1.0 for 12 h and then the levels of protein (B and C) and the TCID_50_ (D and E) were examined. (F) the ubiquitination of the H9N2 WT and four mutant viruses except for R^95^K and K^242^N virus were markedly promoted by TRIM21. Myc-tagged vector or Myc-tagged TRIM21 and HA-tagged Ub-WT were co-transfected into HEK293T cells. After 24h, the cells were infected with H9N2 WT and mutant IAV (H9N2 M1-T^37^A, H9N2 M1-R^95^K, H9N2 M1-S^224^N, H9N2 M1-K^242^N) at an MOI = 1.0 for 12h. The cells were then treated with 25μM MG132 for 6 h before harvest, cell lysates were subjected to IP with anti-M1 and the precipitation was analyzed by western blot with the indicated antibodies. (G-J) The RING domain of TRIM21 is critical for inhibiting H9N2 virus replication. HEK293T cells were transfected with Myc-tagged vector or Myc-tagged TRIM21 WT, C^16^A mutant and ΔRING-deleted TRIM21 plasmids for 24 h, the cells were infected with H9N2 virus at an MOI = 1.0 for 12 h, and then the levels of viral protein (G), mRNA (H), vRNA (I), and the TCID_50_ (J) were examined. Each experiment was independently performed with three biological repeats. All results are presented as means ± SD. *, *p <* 0.05; **, *p <* 0.01; ns, *p >* 0.05.

To investigate whether the mutations T^37^A, R^95^K, S^224^N, and K^242^N are required for TRIM21-mediated inhibition of H9N2 replication, viruses with single amino acid site mutations of M1 (T^37^A, R^95^K, S^224^N, and K^242^N) were rescued in HEK293T cells, which were subsequently inoculated and propagated in 9-day-old SPF embryonated chicken eggs. Then, we infected A549 cells with an equal amount of rescued wild-type and mutant viruses at the initial step, collected supernatants at various time points, and detected viral replication growth properties using TCID_50_ assays in A549 cells, represented by viral titers. The results showed that mutations of R^95^K and K^242^N, but not T^37^A and S^224^N, significantly improved virus proliferation, indicating that the R^95^ or K^242^ is critical for restricting of virus replication (Fig 5A). We next tested the effect of TRIM21 on the replication of these virus mutants by infecting *TRIM21*-overexpressing or *TRIM21*-KO A549 cell lines with the rescued viruses (MOI = 1.0). The results showed that the mutation of R^95^K or K^242^N blunted the effects of TRIM21-mediated inhibition of H9N2 (Fig 5B–5E). Similar results were obtained in *TRIM21*-overexpressing or *TRIM21*-KO HEK293T cell lines infected with the rescued H9N2 M1 mutant viruses (S4A–S4D Fig). Consistently, the H7N9 virus with K^95^R/N^242^K mutation of the M1 protein (H7N9-M1-K^95^R/N^242^K) displayed an opposite result compared to the H9N2 virus with R^95^K mutation (S4E–S4G Fig). These data demonstrate that the R^95^ and K^242^ sites of IAV M1 protein are critical for TRIM21-mediated inhibition of virus replication. Subsequently, when cells were infected with the rescued H9N2 mutant viruses (T^37^A, or R^95^K, or S^224^N, or K^242^N) individually, a decrease in TRIM21-induced ubiquitination was only related to the residues R^95^ and K^242^ of H9N2 M1 protein (Fig 5F), showing that the residues R^95^ or K^242^ of M1 are critical for TRIM21-mediated ubiquitination degradation.

To further verify the E3 ligase activity of TRIM21 that affects H9N2 virus replication, A549 cells were transfected with TRIM21 deletion mutants for the RING (ΔRING) and the C^16^A mutant separately, and then infected with H9N2 virus. The results showed that protein level, *M1* mRNA, vRNA, and viral titer of H9N2 in the TRIM21 mutant ΔRING and C^16^A-transfected cells increased significantly compared to that in wild-type TRIM21-transfected cells (Fig 5G–5J), revealing that the E3 ligase activity of TRIM21 is necessary to inhibit H9N2 replication.

### TRIM21 and the residues R^95^ and K^242^ of M1 are critical for IAV replication *in vivo*

**Fig 6:**
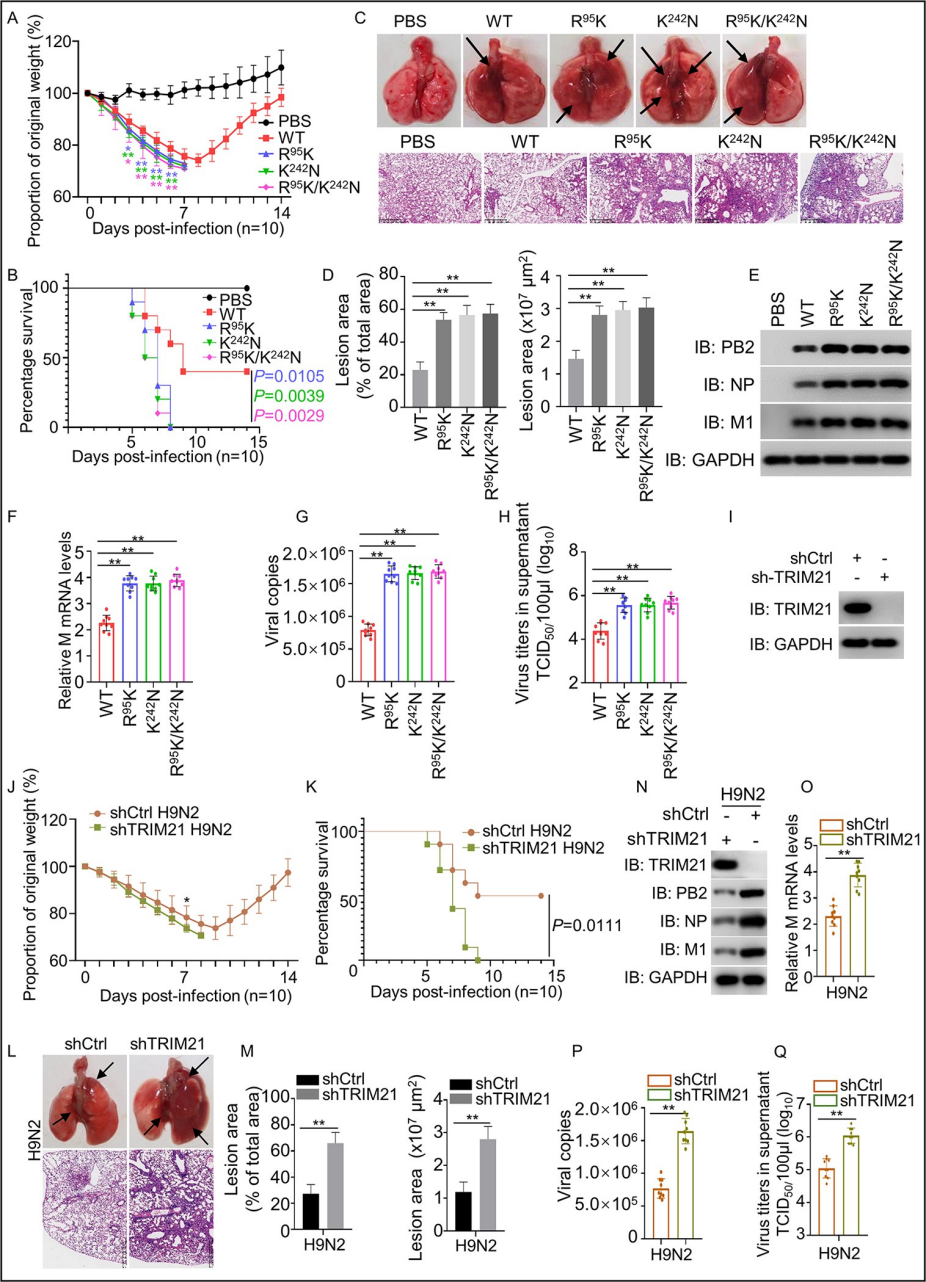
Effect of TRIM21 knockdown and M1 mutation (R^95^K and K^242^N) on pathogenicity in mice. The 8-week-old C57BL/6 mice (13 per group) were intranasally infected with WT H9N2 virus, H9N2 mutant viruses R^95^K, K^242^N, or R^95^K/K^242^N at a dose of 10^7.0^ TCID_50_ H9N2 virus, respectively. In another experiment, the 3-week-old C57BL/6 mice (thirteen per group) were intranasally infected with 10^11.0^ TCID_50_ of AAV6 with the TRIM21-shRNA (shTRIM21) or scrambled shRNA control (shCtrl). Four weeks after infection, the treated mice were intranasally infected with H9N2 (10^7.0^TCID_50_). Three mice per group were euthanized on day 6 post-infection to check for lesions and virus replication in the lungs. The remaining mice (10 per group) were monitored until day 14. Mice with a weight loss of more than 30% of their initial body weight were euthanized and recorded as dead. (A and B) Curves of body weight (A) and survival (B) in mice (n = 10 mice) from 0 to 14 days after infection with WT H9N2 virus or H9N2 mutant viruses R^95^K, K^242^N and R^95^K/K^242^N. (C) Gross and histopathological lesions in the lung at 6 day after infection. (D) The lesion area was measured as a percentage and μm^2^ of the total lung area in (C). (E-H) The lung of infected mice was harvested to detect the levels of viral proteins (E), mRNA level of *M* gene (F), viral RNA copies (G), and the TCID_50_ (H). (I) Validation of shTRIM21 knockdown effect in mice. 3-week-old C57BL/6 mice were intranasally infected with shCtrl or shTRIM21 for four weeks, and TRIM21 expression in mouse lungs was detected by Western blot using the TRIM21 antibody. (J-K) Curves of body weight (J) and survival (K) in *TRIM21* knocked down mice from 0 to 14 days after infection with WT H9N2 virus (n = 10 mice). (L) Gross and histopathological lesions in lungs of *TRIM21* knocked down mice at 6 days after infection. (M) The lesion area was measured as a percentage and μm^2^ of total lung area in (L). (N-Q) The lungs of TRIM21 knocked down mice with H9N2 infection were harvested to detect the levels of viral proteins (N), mRNA level of *M* gene (O), viral RNA copies (P), and the TCID_50_ (Q). Each experiment was independently performed with three biological repeats. All results are presented as means ± SD.*, *p <* 0.05; **, *p <* 0.01; ns, *p >* 0.05. The photo by Lulu Lin.

To further identify the importance of R^95^ and K^242^ of M1 in IAV pathogenesis, we infected 8-week-old C57BL/6 mice with H9N2 WT, R^95^K, K^242^N, R^95^K/K^242^N mutant viruses, respectively. The results showed that mice infected with the R^95^K, K^242^N, and R^95^K/K^242^N mutants lost weight more rapidly than those infected with the WT H9N2, and the mutant virus-infected mice died by the 8^th^ day post-infection, whereas only 60% of the WT virus-infected mice died (Fig 6A and 6B). Consistently, compared to WT H9N2-infected mice, the lungs in the mutant virus-infected mice showed more severe lesions (Fig 6C–6D) and a remarkable enhancement of virus replication (Fig 6E–6H). These results suggested that R^95^ and K^242^ in M1 are the critical residues for enhancing the replication and pathogenicity of IAV in mice.

To investigate the effect of TRIM21 on IAV replication in *vivo*, we performed genetic knockdown in mice lungs using Adeno-associated virus (AAV6) containing the validated *TRIM21* shRNA (AAV6-shTRIM21) and control shRNA (AAV6-shCtrl). Then, 3-week-old C57BL/6 mice were intranasally infected with AAV6-shTRIM21 or AAV6-shCtrl for four weeks, followed by infection with IAV (H3N2, H5N1 and H9N2) for 14 days. Western blot assay showed that TRIM21 expression decreases in shTRIM21 treated mice (Fig 6I). After infection with IAV, shTRIM21-treated mice experienced severe body weight loss and decreased survival compared to shCtrl-treated mice (Figs 6J–6K and S5A–S5D). Similarly, compared to shCtrl-treated mice, the lungs in the shTRIM21-treated mice exhibited more severe lesions and significantly enhanced viral replication (Figs 6L–6Q and S5E–S5L). Overall, these results indicate the important role of TRIM21 in inhibiting virus replication and mitigating IAV pathogenicity in mice.

### The R^95^K mutation of M1 enhances host adaptation of influenza A virus

**Fig 7:**
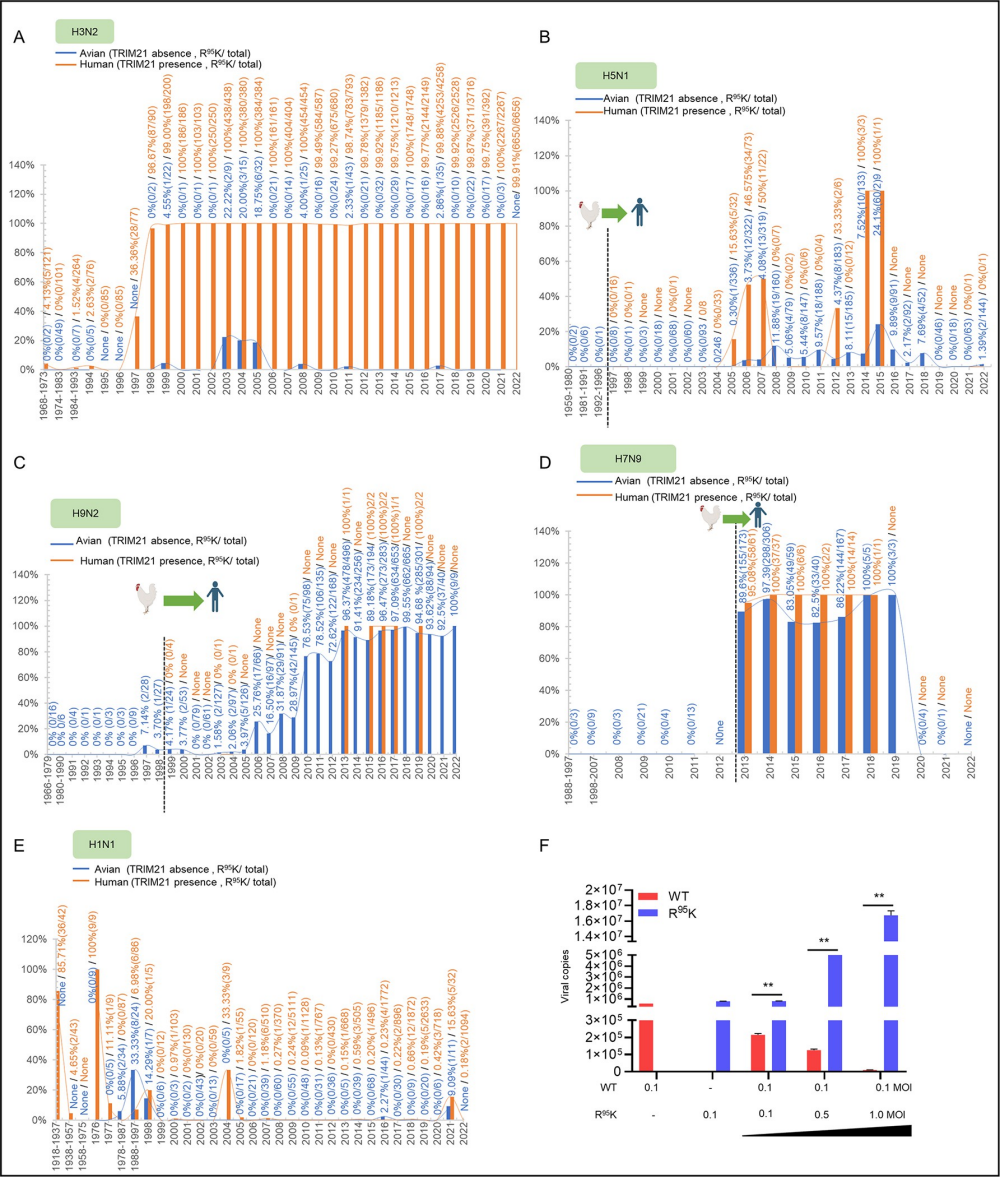
Replication ability of H9N2 virus with or without the residue R^95^ of M1. (A-E) Proportions of the R^95^K mutation in the M1 protein of H3N2 (A), H5N1 (B), H9N2 (C), H7N9 (D), and H1N1 (E) viruses. M1 sequence data of different IAV subtypes were obtained from the NCBI GenBank Database. The image was created using the website https://app.biorender.com/. (F) A549 cells were co-infected with WT (MOI = 0.1) and R^95^K mutant H9N2 viruses (MOI = 0.1, 0.5. and 1.0) for 12 h, and then the vRNA was examined. Each experiment was independently performed with three biological repeats. All results are presented as means ± SD. *, *p <* 0.05; **, *p <* 0.01; ns, *p >* 0.05.

Given the evidence that the R^95^K mutation in the M1 resists TRIM21-mediated ubiquitination and degradation, and enhanced the replication of the R^95^K mutant virus significantly (Figs 5 and 6A–6H), we speculate whether viruses are evolving to escape TRIM21 inhibition while TRIM21 inhibits IAV replication. Then, we investigated the R^95^ mutation in the amino acid sequences of M1 proteins from all H1N1, H3N2, H5N1, H7N9, and H9N2 IAV strains deposited in the GenBank database between 1918 and 2022. The results showed H3N2 and H5N1 AIVs carrying the R^95^K mutation of M1 sustained relatively low in birds but not in humans since they were recorded in 1999 and 2005, respectively (Fig 7A and 7B), suggesting that the residue R^95^ of M1 in H3N2 and H5N1 AIVs is stable in birds. In contrast, the data in Fig 7C showed that the H9N2 AIV in 1966 and H9N2 AIV with the M1 R^95^K mutation recorded in 1997 became gradually the dominant epidemic virus since 2003. In particular, after the outbreak of human H7N9 subtype avian influenza in 2013, the H9N2 virus with the M1 R^95^K mutation in birds maintained a frequency of more than 89%. It is worth noting that H7N9 virus the M1 R^95^K was not detected in humans and birds until the H7N9 virus suddenly jumped to humans in 2013 (Fig 7D), and both H7N9 and H9N2 with the M1 R^95^K were isolated with high frequency (poultry:82.5%-100%; human:95.08%-100%). Fig 7E showed that similar to H3N2 and H5N1, the residue R^95^ of M1 in H1N1 AIVs is stable in birds. Interestingly, early H1N1 PR8 strain (1918–1976) contained M1 with K^95^, which escapes from recognition by TRIM21 as mentioned in our study. However, distinguish from the aforementioned types of IAV (H3N2, H5N1, H7N9, and H9N2), the H1N1 M1 with R^95^ has become dominant since 1977. We conjecture that H1N1 may employ another mechanism to escape from TRIM21-mediating degradation of M1. Based on the above information, to investigate whether the high frequency of virus carrying the R^95^K of M1 is closely related to the replication ability of IAV, A549 cells were co-infected with WT and recombinant H9N2 viruses using the gradient dilution method. In the qRT-PCR assay of the coinfection experiment, transcripts encoding M1 with the R^95^K mutation increase by about four times compared to transcripts encoding M1 with R^95^ (Fig 7F), showing that the H9N2 virus with the M1 R^95^K mutation competitively inhibited the H9N2 virus with the M1 R^95^ (wild type H9N2) and became the predominant circulating virus. These findings suggested that the M1 R^95^K mutation is a host-adapted mutation that enhances host replication of IAV.

## Discussion

The H1N1, H3N2, H5N1, H7N9 and H9N2 are currently the most prevalent IAV subtypes. Among them, seasonal H1N1 and H3N2 viruses primarily infect humans, while H5N1, H7N9, and H9N2 viruses mainly circulate in birds and occasionally infect humans. Thus, studying these subtypes can provide important scientific evidence for the prevention and control of influenza outbreaks. In this study, TRIM21 displayed a direct interaction with M1 from H3N2, H5N1, and H9N2 IAVs but not PR8 and H7N9, and limited their replication (Figs 1, 4, S2 and S3). TRIM family proteins interfere with IAV replication by directly interacting with viral proteins and promoting polyubiquitination-dependent degradation. TRIMs of the same species are composed of distinct domains, whereas TRIMs are relatively conserved among different species [44,45]. The evolutionary conservation of TRIM21 among species further suggests that TRIM21 has similar or identical functions in different species [20]. Several TRIM family proteins directly limit IAV replication by inducing proteasomal degradation of viral proteins, including polymerase subunits PB1 and NP [26,28,29]. Although TRIM21 has been reported to limit the replication of adenoviruses, rhinoviruses, rotaviruses and LCMV [46–50], its role in the regulation of influenza A virus is poorly understood. Thereby, our data demonstrated that TRIM21 is a host restriction factor of infection by certain IAV subtypes, implying that TRIM21 may be a factor that influences the host adaption of IAV.

E3 ubiquitin ligases are known to have specific substrate proteins. Studies have shown that the host E3 ubiquitin ligases can be employed by viral proteins to mediate self-ubiquitination, thereby regulating viral replication. TRIM21 is an E3 ligase and cytosolic Fc receptor for antibody-dependent neutralization and antibody-mediated degradation [35,51]. TRIM21 was identified as the restriction factor of non-enveloped DNA (adenoviruses human adenovirus1 and mouse adenovirus1) or RNA (feline calicivirus) viruses by binding via the PRY/SPRY domain, which catalyzed the formation of K63-linked ubiquitin chains, and stimulated the transcription factor pathways of NFκB, AP1, IRF3, IRF5, and IRF7 [32,52]. Additionally, TRIM21 has been reported to enhance the antiviral response by inhibiting the ubiquitination-dependent degradation of IRF3 [53]. Thus, TRIM21 functions as both a viral restriction factor and a modulator of innate immune signaling. Interestingly, in the present study, we found that the RING domain is critical for the antiviral activity of TRIM21 (Fig 5G–5J). Moreover, the residues R^95^ and K^242^ in M1 serve as the binding sites and the ubiquitination site for TRIM21 (Figs 2 and 3), respectively. Taken together, we revealed that the TRIM21 binding to the residue R^95^ of M1 facilitates K48 ubiquitination of M1 K^242^ for proteasome-dependent degradation, leading to the inhibition of H3, H5, and H9 IAV replication (Figs 2–4 and S1–3). Additionally, we found that the replication levels of both R^95^K and K^242^N mutant viruses were lower than that of WT H9N2 virus in *TRIM21*-KO cells, although the replication ability of R^95^K and K^242^N mutant viruses was improved compared with WT H9N2 (Fig 5E), indicating that R^95^K and K^242^N mutant viruses may have intrinsic defects. Given the importance of M1 in progeny virion budding [11,12], we found that the residue substitutions R^95^-to-K or K^242^-to-N might affect budding process (S6 Fig). These findings suggest that the ubiquitination pathway of IAV with the M1 R^95^/K^242^, as an enveloped RNA virus, is different from that of non-enveloped DNA and RNA viruses. However, whether TRIM21 inhibits the replication of certain IAV subtypes by promoting innate immunity needs further investigation in the future.

The host-adaptive mutations of viruses are important for infection. Influenza viruses have evolved with multiple layers of adaptive strategies to evade these restrictions. PB2 host-adaptive mutations might be associated with the evasion of the antiviral effects of the autophagy process [54] and were associated with the production of new virions [55]. Host-adaptive mutations of K^78^/K^79^ in M2 were caused by the inhibitory pressure of membrane associated ring-CH-type finger 8 [56]. Influenza A virus with the R^95^K mutation in M1 has been reported to be associated with airborne transmission [43]. In the present study, we have discovered that R^95^K mutation primarily occurs in avian flu strains that incidentally infect humans, such as H5N1, H7N9, H9N2 (Fig 7). This mutation allows avian flu to evade the human TRIM21-mediated degradation system, thereby facilitating robust replication in human cells. Interestingly, the mutation does not appear to be required when the virus is circulating in birds, as TRIM21 is absent in avian. However, the H1N1 actively replicates in humans did not show the mutation at R^95^, the exact reason for this remains unclear. One possibility is that H1N1 may employ another mechanism to evade TRIM21-mediated degradation of the M1 protein. Previous report has suggested that AIMP2-mediated competition for ubiquitination at K^242^ of M1 by SUMOylation precluded the proteasome-mediated degradation of M1 [57]. Thus, based on the evidence presented above, we believe that during the co-evolution of the virus and host, the residue R^95^ in M1 of certain subtypes of IAV would evolve into K to evade host restriction for self-replication in the presence of TRIM21.

Overall, our study reveals the role of TRIM21 as a host restriction factor for IAV. Mechanistically, TRIM21 promotes proteasome degradation of M1 by interacting with R^95^ of M1 and subsequently the K48-linked polyubiquitination at K^242^, and the mutation at R^95^ of IAV M1 allows it to escape TRIM21-triggered proteasome degradation in mammals, implying TRIM21 in mammals is one of driving forces behind virus evolution.

## Materials and methods

### Ethics statement

All experiments on H5N1 and H7N9 were conducted in Institute of Animal Husbandry and Veterinary, Fujian Academy of Agricultural Sciences. This study was approved by Animal Care and Use committee of the Institute of Animal Husbandry and Veterinary Medicine, Fujian Academy of Agriculture Sciences. All procedures were carried out in accordance with the regulations and policies governed by the committee (Approval No. 202208001).

### Cell, virus, plasmids and animals

HEK293T cells (Human embryonic kidney cell line, ATCC, CRL-11, 268) and A549 cells (Human non-small cell lung cancer cell line, CRM-CRL-185) were purchased from American Type Culture Collection (ATCC). HEK293T cells and A549 cells were cultured in Dulbecco’s modified Eagle medium (DMEM; Gibco, Carlsbad, CA USA) with 10% fetal bovine serum (FBS; 1616756, Biological Industries, Israel). A/Puerto Rico/8/34(PR8), A/swine/Guangdong/04 (H3N2), A/Chicken/Jiande/09/2009 (H9N2) and A/Hangzhou/1/2013(H7N9) were stored in our laboratory [58–61]. The A/Duck/Fujian/2018 (H5N1) were reserved in Fujian Academy of Agricultural Sciences Institute of Animal Husbandry and Veterinary medicine, China. AAV6 (10^11.0^ TCID_50_) with the TRIM21-shRNA (shTRIM21) or scrambled shRNA control (shCtrl) was purchased from WZ Biosciences Inc. (Shangdong, China). The 3-week-old and 8-week-old C57BL/6 background mice were purchased from Shanghai Slac Co. Ltd. (Shanghai, China).

### Antibodies and reagent

Rabbit polyclonal antibody (pAb) against Myc (R1208-1), GAPDH (glyceraldehyde-3-phosphate dehydrogenase; R1210-1), His (0812–1) and mouse mAbs against GST (M0807-1) were purchased from Huaan Biological Technology (Hangzhou, China). Mouse anti-Myc (05–419), anti-Flag (F1804) and anti-HA (H3663) mAbs for IP as well as chloroquine phosphate (CQ; 1118000) were purchased from Sigma-Aldrich (St. Louis, MO, USA). Rabbit mAbs against TRIM21 (12108-1-AP) was purchased from Proteintech Group, Inc. (Manchester, UK). Mouse mAbs to PB2, NP, M1 and M2 were produced by our laboratory [62]. Horseradish peroxidase (HRP)-labeled goat anti-mouse or anti-rabbit IgG was purchased from KPL (Milford, MA, USA). Anti-mouse IgG HCS HRP-linked antibody and anti-Rabbit IgG LCS HRP-linked antibody (A25112 and A24022) were purchased from ABBkine Scientific (Wuhan, China). ChamQ Universal SYBR qPCR Master Mix (Q711-02) was purchased from Vazyme (Nanjing, China). NP-40 cell lysis buffer (P0013F) and MG132 (S1748) were purchased from Beyotime (Shanghai, China). MG132 was solubilized in Dimethyl sulfoxide (DMSO) to a stock of 25μM. PMSF (P0100) was purchased from Solarbio Biological Technology (Beijing, China). Protein A/G plus agarose (sc-2003) was purchased from Santa Cruz Biotechnology. GST resin (16100) was purchased from Thermo Fisher (Waltham, MA, USA).

### DNA construction and transfection

Vectors Flag-gst-vc constructed and stored in our laboratory [63]. The M1 cDNA fragments from A/Puerto Rico/8/34(PR8), A/swine/Guangdong/04 (H3N2), A/Duck/Fujian/2018 (H5N1), A/Hangzhou/1/2013(H7N9) and A/Chicken/Jiande/09/2009 (H9N2) were cloned separately into vectors pCMV-Myc-N (635689; Clontech, Mountain View, CA, USA), pCMV-Flag-N (635688; Clontech, Mountain View, CA, USA) pCMV-Flag-N-GST, pET-28a-C (69864–3; Novagen, Madison, WI, USA) for different uses. The resulting plasmids were named Flag-PR8/H3/H5/H7/H9-M1, Flag-gst-PR8/H3/H5/H7/H9-M1, Myc-H9-M1 and His-H9-M1. The M1 segment of H9N2 virus with M1-T^37^A, R^95^K, S^224^N and K^242^N mutant was generated by site-directed mutagenesis in the vRNA-mRNA bidirectional transcription vector pLLBA. Several Myc-H9-M1, Flag-gst-H3-M1, Flag-gst-H5-M1, Flag-gst-H9-M1, pLLBA-H9-M1 mutants were created using site-specific mutagenesis. The M1 segment of H7N9 virus with M1-K^95^R/N^242^K mutant was generated by site-directed mutagenesis in the vRNA-mRNA bidirectional transcription vector pLLBA. The full-length cDNA sequences of TRIM21 was amplified from HEK293T cells and cloned into vectors pCMV-Myc-N, pGEX-4T-1(27-4580-01; GE healthcare, Chicago, IL, USA), pCDH-EF1-MCS-T2A-Puro (CD520A-1, System Biosciences) using specific primers. The resulting plasmids were Myc-TRIM21, GST-TRIM21, pCDH-TRIM21. The deletion mutants ΔRING, ΔBB, ΔCC and ΔPRY/SPRY were cloned using deletion primers from TRIM21. The primers used for the construction of the TRIM21, H3N2 M1, H5N1 M1, H7N9 M1 and H9N2 M1 mutants are listed in S1 Table. Plasmids HA-Ub-WT, HA-Ub-K48 and HA-Ub-K63 were kindly provided by Hongbin Shu [64].

### Preparation of cell samples for mass-spectrometry

A549 cells infected with H9N2 virus (MOI = 1.0) were collected and lysed using NP40 lysis buffer (P0013F, Beyotime, Beijing, China) supplemented with PMSF (P0100, Solarbio, Beijing, China). Cellular lysates were incubated with mouse anti-M1 mAb or IgG as the control for 4h, followed by incubation with protein A/G beads (sc-2003, Santa Cruz Biotechnology, CA, USA) for 4h at 4°C. The samples were centrifuged and supernatants were removed. Then, the pellets were resuspended with NP40 as a wash buffer, repeated five times. Finally, the pellets were sent to company (Applied Protein Technology, China) for further analysis by mass-spectrometry.

### Virus infection and titer determination

HEK293T cells or A549 cells were infected with PR8, H3N2, H5N1, H7N9, H9N2 at different time points after infection, the cells were then harvested and freeze-thawed three times. After centrifugation at 12,000*×g* for 10 min at 4°C, the supernatants were used for 50% tissue culture infective dose (TCID_50_) detection. The viral suspension was diluted in DMEM and incubated for 1h. Then the DMEM was changed to DMEM with 2% FBS and 1 μg/ml N-p-tosyl-L-phenylalanine chloromethyl ketone (TPCK)-treated trypsin (LS003750, Worthington Biochemicals) for 72 h. The TCID_50_ assay was measured with eight parallels dilution and a control group to determine the virus titer. The infected cells were subjected to an IFA assay using anti-M2 mouse mAbs and observed using a fluorescence microscope (OLYMPUS, U-REL-T). Finally, the data were calculated according to the Reed-Muench method.

### Generation of TRIM21 knockout HEK293T cells and A549 cells

The TRIM21 gene target sequence, 5’-TCATCTCAGAGCTAGATCGA-3’, was inserted into plasmid lentiCRISPR v2 (Addgene, 52961, depositing lab: Feng Zhang from Broad Institute). The constructed *TRIM21*-KO and lentiCRISPR v2 as a control were transfected into HEK293T cells and A549 cells for 48h, respectively, and then selected under puromycin (10μg/ml) for 72h. For colony formation, the selected cells were diluted to 50 cells/ml and inoculated into 96-well plates. Until the colony formation, each colony was separately transferred into 48-well plates. Finally, the TRIM21 knockout cell lines were identified by sequencing and Western blot.

### Construction of A549 cell lines with stable expression of *TRIM21*

The *TRIM21* gene was cloned into overexpression lentivirus vector pCDH-CMV-MCS-EF1-Puro (System Biosciences, CD510B-1). Then, the recombinant construct and ViraPower lentiviral Packaging Mix (psPAX2 (12260; Addgene) and pMD2.G (12259; Addgene) were co-transfected into HEK293T cells for 48 h in a 4:3:1 proportion, with an empty vector as a control. The cell supernatants containing viral particles were harvested and used to infect A549 cells. At 24 h after infection, the cells were passaged and selected using 10μg/ml puromycin. The expression of TRIM21 protein in the A549 cell lines was detected by immunoblotting.

### RT-qPCR

HEK293T cells and A549 cells were infected with PR8, H3N2, H5N1, H7N9, H9N2 (MOI = 1.0) for 12h, and the infected cells were collected. The total RNA of whole cellular lysates was isolated with RNAiso Plus (9109; Takara, Shiga, Japan) according to the manufacturer’s instructions. DNase I (M0303, NEB, New England Biolabs, USA) was used to remove DNA. RevertAid RT reverse transcription kit (K1691, Thermo Fisher, MA, USA) was used for reverse transcription according to the manufacturer’s instructions. The relative abundance of transcripts was measured using ChamQ Universal SYBR qPCR Master Mix (Q711-02; Vazyme, Nanjing, China), the LightCycler 96 sequence detector system (Roche), and the primer as described previously [63]. The primers for qRT-PCR are listed in S1 Table.

### Co-immunoprecipitation (Co-IP)

The experiment was performed as previously described [63]. Briefly, the HEK293T cells were co-transfected with the indicated plasmids for 48h. Then, the resulting cells were collected and lysed using NP40 lysis buffer containing PMSF for 4 h at 4°C. Cellular lysates were centrifuged at 12,000*×g* for 10 min and incubated with indicated antibody for 4 h, followed by incubation with protein A/G beads for 4h at 4°C. After centrifugation at 1000*×g* for 5 min at 4°C. The supernatants were removed, and the pellets were resuspended with NP40 as a wash buffer and repeated five times. Finally, the pellets were lysed in lysis buffer for Western blot.

### Immunofluorescence assay and confocal microscopy

These processes were previously described [65]. HEK293T or A549 cells grown on glass-bottom dishes or 96-well cell culture plates were transfected and/or infected as indicated. The samples were fixed in 4% paraformaldehyde for 15 min at room temperature, and permeabilized with 0.1% Triton-X-100 for 10 min. The permeabilized cells were blocked with 5% skim milk for 1 h, and then stained with the indicated primary antibodies for 2 h at 37°C. After being washed three times with PBS, samples were incubated with secondary antibodies conjugated to Alexa Fluor 488 (A21210, Invitrogen, USA) or Alexa Fluor 546 (A10036, Invitrogen, USA) for 1 h at 37°C. Nuclei were counterstained with DAPI (4’,6-diamidino-2-phenylindole) for 10 min. The cells were scanned with an LSM780 laser scanning confocal microscope (Zeiss, Oberkochen, Germany).

### GST pull down assay

GST-TRIM21 was expressed from pGEX-4T-1 vector and pET-28a-C-His-H9-M1 in *Escherichia coli* BL21 (Sangon Biotechnology, B528415-0010) by induction with 0.1 mM isopropyl-1-thio-β-D-galactopyranoside (IPTG) (Sangon Biotechnology, A600168-0025) at 16°C overnight. The GST and GST-TRIM21 were purified with Pierce Glutathione Agarose (16100, Thermo Fisher, MA, USA) according to the manufacturer’s protocol. His-H9-M1 was purified with Ni-NTA agarose resins (163014747; QIANGEN, Hilde, Germany) according to the manufacturer’s protocol. The purified GST (10μg) or GST-TRIM21 (10μg) was incubated with His-H9-M1(20μg) for 4 h at 4°C and then added with Pierce Glutathione Agarose incubated for 4 h at 4°C. The bound proteins were detected by western blot.

### Ubiquitin assays

To analyze the effect of TRIM21 on the ubiquitination of M1 in HEK293T cells. The Myc-tagged vector or TRIM21 was co-transfected with Flag-gst-tagged PR8/H3/H5/H7/H9 M1 or its mutants and HA-tagged Ub-WT or its mutants into HEK293T cells for 48h, followed by treatment with 25μM MG132 for 6 h and lysed with NP40 containing 6M urea and 1 mM PMSF for 4 h at 4°C. The lysates were then centrifuged at 12,000×*g* for 10 min at 4°C, the supernatants were diluted with NP40 and added to the Pierce Glutathione Agarose for 4 h at 4°C, the Pierce Glutathione Agarose was washed five times with NP40 containing 0.5 mM Dithiothreitol (DTT) (Sangon Biotechnology, A100281), and the supernatant was removed. Finally, the Pierce Glutathione Agarose was lysed with lysis buffer for western blotting.

### Generating mutant IAV

The recombinant WT or mutant viruses with M1 site mutations, H9N2 M1-WT, rH9N2, H9N2 M1-T^37^A, H9N2 M1-R^95^K, H9N2 M1-S^224^N, H9N2 M1-K^242^N, H9N2 M1-R^95^K/K^242^N, H7N9 M1-WT, H7N9 M1-K^95^R/N^242^K were generated using the reverse genetic system as previously described [66]. Briefly, the M segment of H9N2 virus with T^37^A, R^95^K, S^224^N, K^242^N, R^95^K/K^242^N mutants and H7N9 virus with K^95^R/N^242^K mutant were generated by site-directed mutagenesis into pLLBA vector. The mutant plasmids were sequenced for confirmation. The viruses were generated by transfecting HEK293T cells with pLLBA-H9/H7-PA, pLLBA-H9/H7-PB2, pLLBA-H9/H7-PB1, pLLBA-H9/H7-NP, pLLBA-H9/H7-NA, pLLBA-H9/H7-HA, pLLBA-H9/H7-NS, pLLBA-H9/H7-M or its mutants co-transfected in DMEM with 1 μg/ml TPCK-treated trypsin (LS003750, Worthington Biochemicals) for 48 h. The H9N2, mutant H9N2 (rH9N2, T^37^A, R^95^K, S^224^N, K^242^N, R^95^K/K^242^N), H7N9, mutant H7N9 (K^95^R/N^242^K) viruses were inoculated into and propagated in 9-day-old SPF embryonated chicken eggs for 3 days and then harvested and stored at -80°C until use.

### Virus growth curve

To further verify that the role of TRIM21 in the key amino acid sites of M1, H9N2 M1-WT, rH9N2, H9N2 M1-T^37^A, H9N2 M1-R^95^K, H9N2 M1-S^224^N, H9N2 M1-K^242^N, H9N2 M1-R^95^K/K^242^N, H7N9 M1-WT, H7N9 M1-K^95^R/N^242^K were rescued as indicated description. Then, A549 cells were respectively infected with an equal amount of H9N2 M1-WT, rH9N2, H9N2 M1-T^37^A, H9N2 M1-R^95^K, H9N2 M1-S^224^N, H9N2 M1-K^242^N, H9N2 M1-R^95^K/K^242^N, H7N9 M1-WT, H7N9 M1-K^95^R/N^242^K virus (MOI = 0.01), and the supernatant was collected at 24 h, 36 h, 48 h and 60 h. The samples were subjected to freeze-thawed three times, and viral titers were determined. The TCID_50_ was calculated to draw viral growth curves.

### Transmissible electron microscopy

Equal amounts of H9N2-WT and mutant viruses (H9N2-R^95^K and H9N2-K^242^N) were used to infect *TRIM21*-KO HEK293T cell lines. The infected cells were scraped from the plates, fixed with 2.5% glutaraldehyde in phosphate buffer (0.1M, pH7.0) for 4h, and then postfixed with 1% OsO4 in phosphate buffer for 1–2 h. The fixed cells were washed three times in the phosphate buffer (0.1M, pH7.0) for 15 min and subsequently dehydrated in a gradient ethanol. They were then placed in Eppendorf containing Spurr resin and heated at 70°C for more than 9 h. The embedded specimen was sectioned using a LEICA EM UC7 ultratome. The sections were stained with uranyl acetate and alkaline lead citrate for 5 to 10 min respectively, and observed using a Hitachi Model H-7650 TEM.

### Mice infection

The 8-week-old C57BL/6 mice (thirteen per group) were intranasally infected with 10^7.0^ TCID_50_ of H9N2 virus (WT or its mutant viruses R^95^K, K^242^N or R^95^K/K^242^N) and ten mice per group were monitored daily for weight loss and clinical sign until 14th day. Mice with a weight loss of exceeding 30% of their initial body weight were euthanized and recorded as dead. Three mice per group were euthanized on day 6 post-infection to check for lesions and virus replication in the lungs. For histopathologic analysis, the lungs were removed on day 6 post-infection and fixed with 4% PFA, embedded in paraffin, sectioned, stained with hematoxylin and eosin solution (H.E). Mice were maintained under specific-pathogen-free conditions in an environment of 20–26°C with 30%-70% humidity and food and water provided ad libitum.

In another experiment, the 3-week-old C57BL/6 mice were intranasally infected with 10^11.0^ TCID_50_ of AAV6 with the TRIM21-shRNA (shTRIM21) or scrambled shRNA control (shCtrl). Four weeks after infection, the treated mice were intranasally infected with H3N2 (10^6.5^ TCID_50_), H5N1 (10^6.7^TCID_50_) and H9N2 (10^7.0^TCID_50_) (thirteen per group) and ten mice per group were monitored daily for weight loss until 14th day. Three mice per group were euthanized on day 6 post-infection to check for lesions and virus replication in the lungs. For histopathologic analysis, the lungs were removed on day 6 post-infection and fixed with 4% PFA, embedded in paraffin, sectioned, stained with hematoxylin and eosin solution (H.E). Mice were maintained under specific-pathogen-free conditions in an environment of 20–26°C with 30%-70% humidity and food and water provided ad libitum.

All animal care and experimental procedures were performed in accordance with the Animal Research Committee guidelines of Zhejiang University (No. ZJU20170667).

### Statistical analysis

The amino acid sequence alignments of M1 protein from H1N1, H3N2, H5N1, H7N9, H9N2 IAVs deposited in GenBank database were performed using MEGA version 7.0. The three-dimensional structural model of M1 protein was performed using PyMOL software. The data were analyzed using GraphPad Prism software 8.0 by a Student’s test (*, *p* <0.05; **, *p* < 0.01; ns, *p* > 0.05). Each experiment was independently performed with three biological repeats. All results are presented as means ± SD.

## Supporting information

S1 Fig K^242^ in M1 of H9N2 virus is required for TRIM21-mediated ubiquitination. (A) All mutants except K^242^R were degraded by TRIM21. Myc-tagged vector or Myc-tagged TRIM21 and Myc-tagged H9N2 M1 WT or arginine mutants were co-transfected into HEK293T cells, and the proteins in the cell lysates were detected using the indicated antibodies. (B) H9N2 M1 K^242^R could not be ubiquitinated. Myc-tagged vector, Myc-tagged TRIM21, HA-tagged Ub-WT, and Flag-GST tagged H9N2 M1 WT or arginine mutants were co-transfected into HEK293T cells for 48 h, followed by treatment with 25μM MG132 for 6 h and the cell lysates were then subjected to immunoprecipitation and western blotting using the indicated antibodies. Each experiment was independently performed with three biological repeats.
(TIF)

S2 Fig TRIM21 inhibits the replication of H5N1 and H3N2 virus rather than H7N9 virus. (A-D) *TRIM21*-expressing A549 cells were infected separately with H7N9, H5N1 and H3N2 at an MOI = 1.0 for 12 h, and then the levels of protein (A), mRNA (B), vRNA (C), and the TCID_50_ (D) were examined. Wild-type A549 cells were used as the control (*, *p <* 0.05; **, *p <* 0.01; ns, *p >* 0.05). (E-H) *TRIM21*-KO A549 cells were infected separately with H7N9, H5N1 and H3N2 at an MOI = 1.0 for 12 h, and then the levels of protein (E), mRNA (F), vRNA (G), and the TCID_50_ (H) were examined. Wild-type A549 cells were used as the control. Each experiment was independently performed with three biological repeats. All results are presented as means ± SD. *, *p <* 0.05; **, *p <* 0.01; ns, *p >* 0.05.
(TIF)

S3 Fig Effects of TRIM21 on the replication of different subtypes of influenza virus in HEK293T cells. (A-D) TRIM21 inhibits the replication of H3N2, H5N1 and H9N2 influenza viruses in HEK293T cells, but has no effect on H7N9 and PR8. HEK293T cells were transfected with Myc-tagged vector or Myc-tagged TRIM21 for 24 h, the cells were then infected with H5N1, H7N9, H3N2, H9N2, PR8 at an MOI = 1.0 for 12 h, and the levels of viral proteins (A), *M1* mRNA (B), vRNA (C), and the TCID_50_ (D) were examined. (E-H) TRIM21 increases the replication of H3N2, H5N1 and H9N2 influenza viruses in *TRIM21*-KO HEK293T cell lines. *TRIM21*-KO HEK293T cell lines were infected with H5N1, H7N9, H3N2, H9N2, and PR8 at an MOI = 1.0 for 12 h, and the levels of protein (E), *M1* mRNA (F), vRNA (G), and TCID_50_ (H) were examined. Each experiment was independently performed with three biological repeats. All results are presented as means ± SD.*, *p <* 0.05; **, *p <* 0.01; ns, *p >* 0.05.
(TIF)

S4 Fig R^95^ or K^242^ sites of M1 are critical for TRIM21-mediated inhibition of H9N2 virus replication in HEK293T cells. (A-D) TRIM21 restricted the replication of the WT and mutant viruses, except for the R^95^K and K^242^N viruses, in HEK293T cells. (A and C) HEK293T cells were transfected with Myc-tagged vector or Myc-tagged TRIM21 for 24 h, the cells were infected with WT H9N2 and mutant H9N2 (T^37^A, R^95^K, S^224^N, K^242^N) at an MOI = 1.0 for 12 h, and then the levels of viral proteins and viral titer were detected using western blotting and TCID_50_, respectively. (B and D) *TRIM21*-KO HEK293T cell lines were infected with WT H9N2 and mutant H9N2 (T^37^A, R^95^K, S^224^N, K^242^N) at a MOI = 1.0 for 12 h, and then the levels of proteins and viral titer were detected using western blotting and TCID_50_, respectively_._ (E) One-step growth curve of WT (H7N9-M1-WT) and mutant H7N9 virus (H7N9-M1-K^95^R/N^242^K). A549 cells were infected with H7N9-M1-WT and mutant H7N9-M1-K^95^R/N^242^K at an MOI = 0.01 for 24 h, 36 h, 48 h and 60 h, respectively, and TCID_50_ was measured as described in the Materials and Methods. (F-G) TRIM21 has no difference on H7N9-M1-WT replication in *TRIM21*-overexpressing and KO A549 cell lines. *TRIM21*-overexpressing, *TRIM21*-KO, and control A549 cells were infected with H7N9-M1-WT and H7N9-M1-K^95^R/N^242^K mutant viruses at an MOI = 1.0 for 12 h, and then the viral titer was detected using TCID_50_. Each experiment was independently performed with three biological repeats. All results are presented as means ± SD. *, *p <* 0.05; **, *p <* 0.01; ns, *p >* 0.05.
(TIF)

S5 Fig TRIM21 inhibited IAV H3N2 and H5N1 replication in mice. The 3-week-old C57BL/6 mice (thirteen per group) were intranasally infected with 10^11.0^ TCID_50_ of AAV6 with the TRIM21-shRNA (shTRIM21) or scrambled shRNA control (shCtrl). At four weeks after infection, the treated mice were intranasally infected with H3N2 (10^6.5^ TCID_50_) and H5N1 (10^6.7^TCID_50_). On day 6 post-infection, three mice per group were euthanized to check for lung lesions and virus replication, while the remaining ten mice per group were monitored until day 14. Mice with a weight loss exceeding 30% of their initial body weight were euthanized and recorded as dead. (A-D) Curves of body weight (A-B) and survival (C-D) in mice (mean ± SD; n = 10 mice) from day 0 to day 14 post-infection. (E-F) Gross (E) and histopathological (F) lesions in the lungs on day 6 post-infection. H&E staining was performed on lung sections (mean ± SD; n = 3 mice). (G-H) The lesion area was measured as a percentage and μm^2^ of the total lung area in (E-F). (I-L) The lungs were harvested to detect the levels of viral proteins (I), mRNA (J), vRNA (K), and the TCID_50_ (L) (mean ± SD; n = 3 mice). Each experiment was independently performed with three biological repeats. All results are presented as means ± SD. *, *p <* 0.05; **, *p <* 0.01; ns, *p >* 0.05. The photo by Lulu Lin.
(TIF)

S6 Fig R95K and K242N mutations affect budding process. *TRIM21*-KO HEK293T cell lines were infected with H9N2-WT virus and mutant viruses (H9N2-R^95^K and H9N2-K^242^N) at an MOI of 20 for 10 h, and the cells were analyzed using transmissible electron microscopy.
(TIF)

S1 Table The primers used for cloning and quantitative real-time PCR. (DOCX)

S1 Data Excel spreadsheet containing, in separate sheets, the underlying numerical data and statistical analysis for Fig panels 3A, 4B-4D, 4F-4K, 5A, 5D-5E, 5H-5J, 6A, 6D, 6F-6H, 6J, 6M, 6O-6Q, 7F, S2B-S2D, S2F-S2H, S3B-S3D, S3F-S3H, S4C-S4G, S5A-S5B, S5G-S5H, and S5J-S5L. (XLSX)
