# Supplementary figures and images for: TRIM21 restricts influenza A virus replication by ubiquitination-dependent degradation of M1
Source: PLoS Pathog. 2023 Jun 21;19(6):e1011472. doi: 10.1371/journal.ppat.1011472 (PMC10325077; doi:10.1371/journal.ppat.1011472)

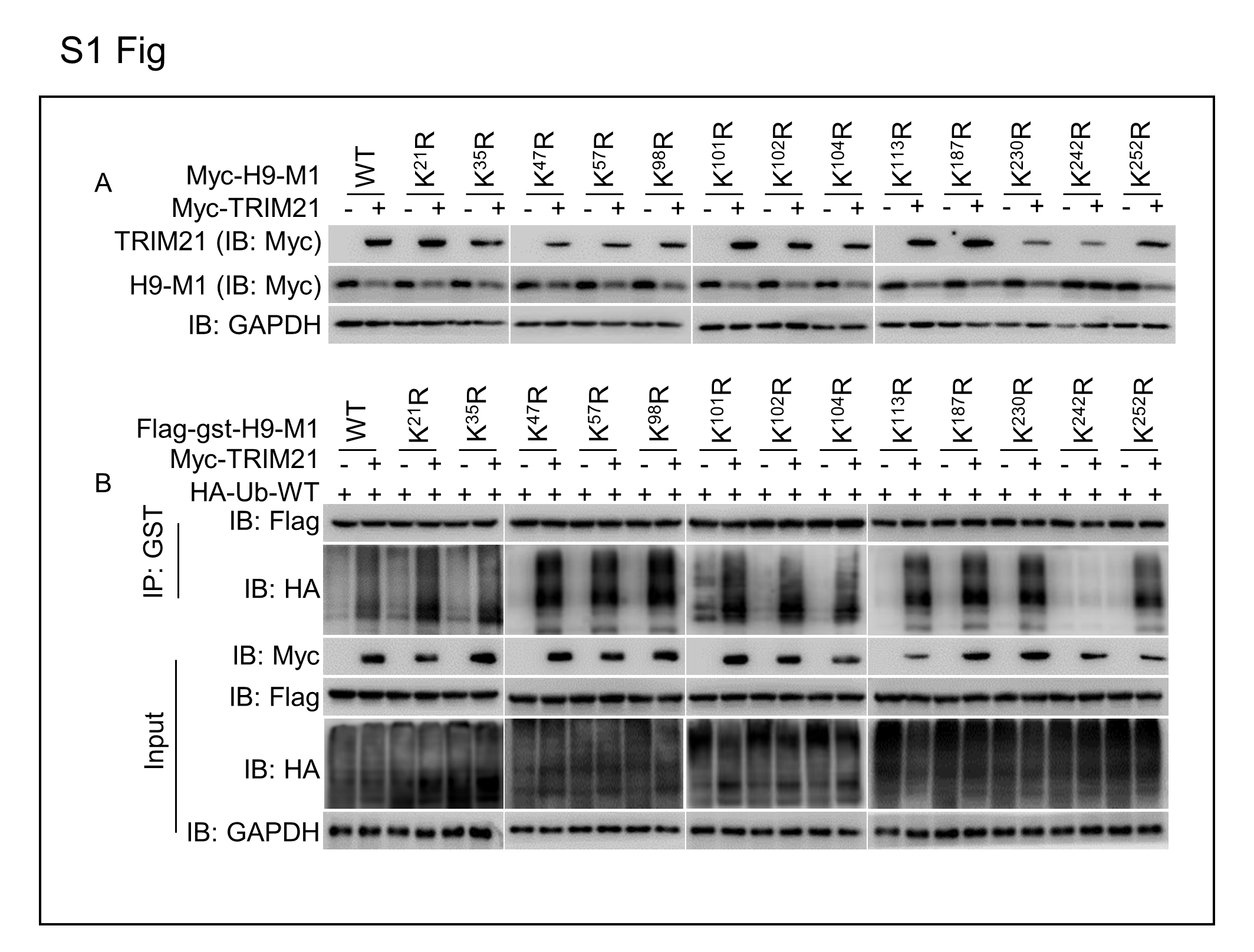

Supplement: S1 Fig — (A) All mutants except K242R were degraded by TRIM21. Myc-tagged vector or Myc-tagged TRIM21 and Myc-tagged H9N2 M1 WT or arginine mutants were co-transfected into HEK293T cells, and the proteins in the cell lysates were detected using the indicated antibodies. (B) H9N2 M1 K242R could not be ubiquitinated. Myc-tagged vector, Myc-tagged TRIM21, HA-tagged Ub-WT, and Flag-GST tagged H9N2 M1 WT or arginine mutants were co-transfected into HEK293T cells for 48 h, followed by treatment with 25μM MG132 for 6 h and the cell lysates were then subjected to immunoprecipitation and western blotting using the indicated antibodies. Each experiment was independently performed with three biological repeats. (TIF) [file ppat.1011472.s001.tif]

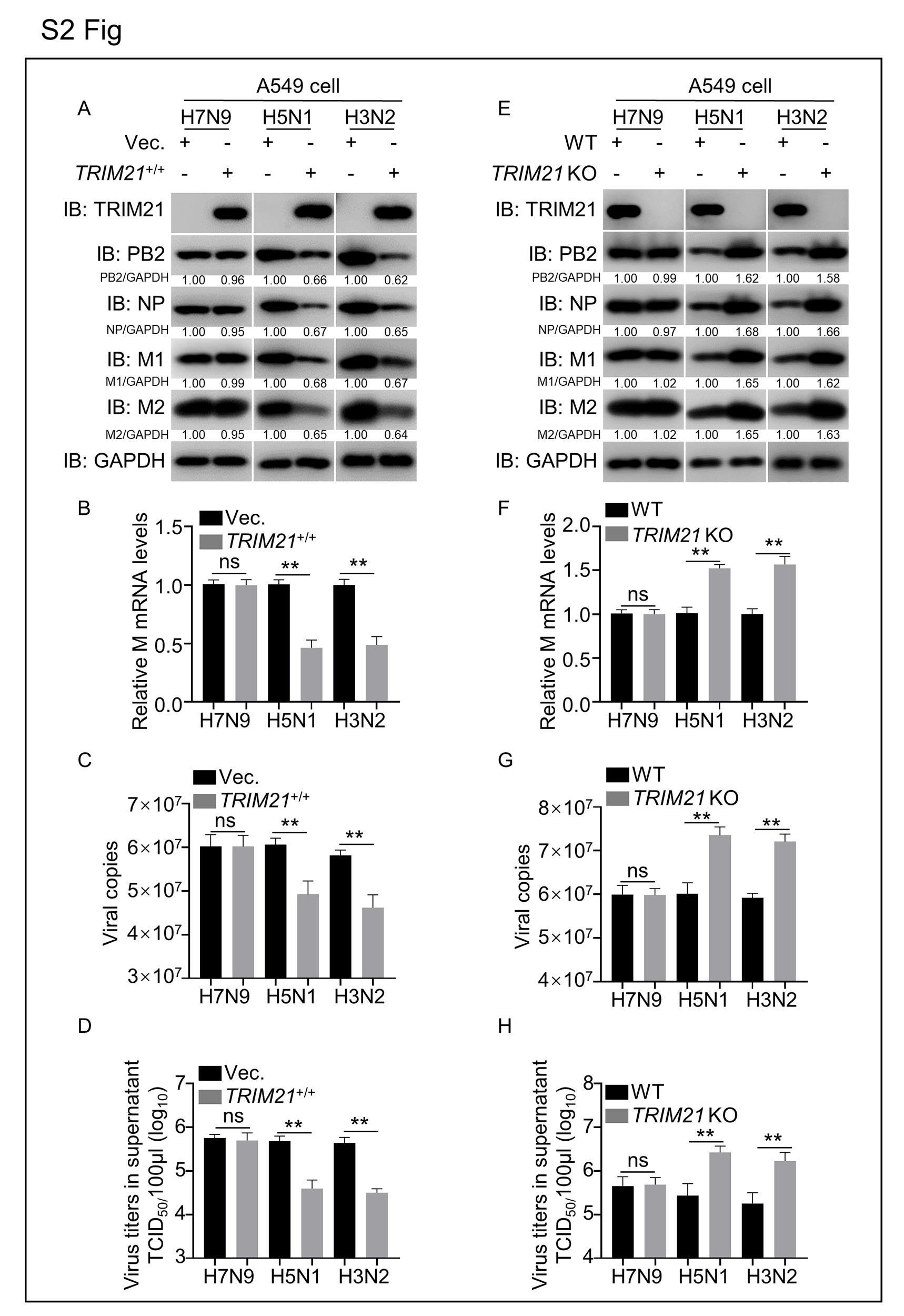

Supplement: S2 Fig — (A-D) TRIM21-expressing A549 cells were infected separately with H7N9, H5N1 and H3N2 at an MOI = 1.0 for 12 h, and then the levels of protein (A), mRNA (B), vRNA (C), and the TCID50 (D) were examined. Wild-type A549 cells were used as the control (*, p < 0.05; **, p < 0.01; ns, p > 0.05). (E-H) TRIM21-KO A549 cells were infected separately with H7N9, H5N1 and H3N2 at an MOI = 1.0 for 12 h, and then the levels of protein (E), mRNA (F), vRNA (G), and the TCID50 (H) were examined. Wild-type A549 cells were used as the control. Each experiment was independently performed with three biological repeats. All results are presented as means ± SD. *, p < 0.05; **, p < 0.01; ns, p > 0.05. (TIF) [file ppat.1011472.s002.tif]

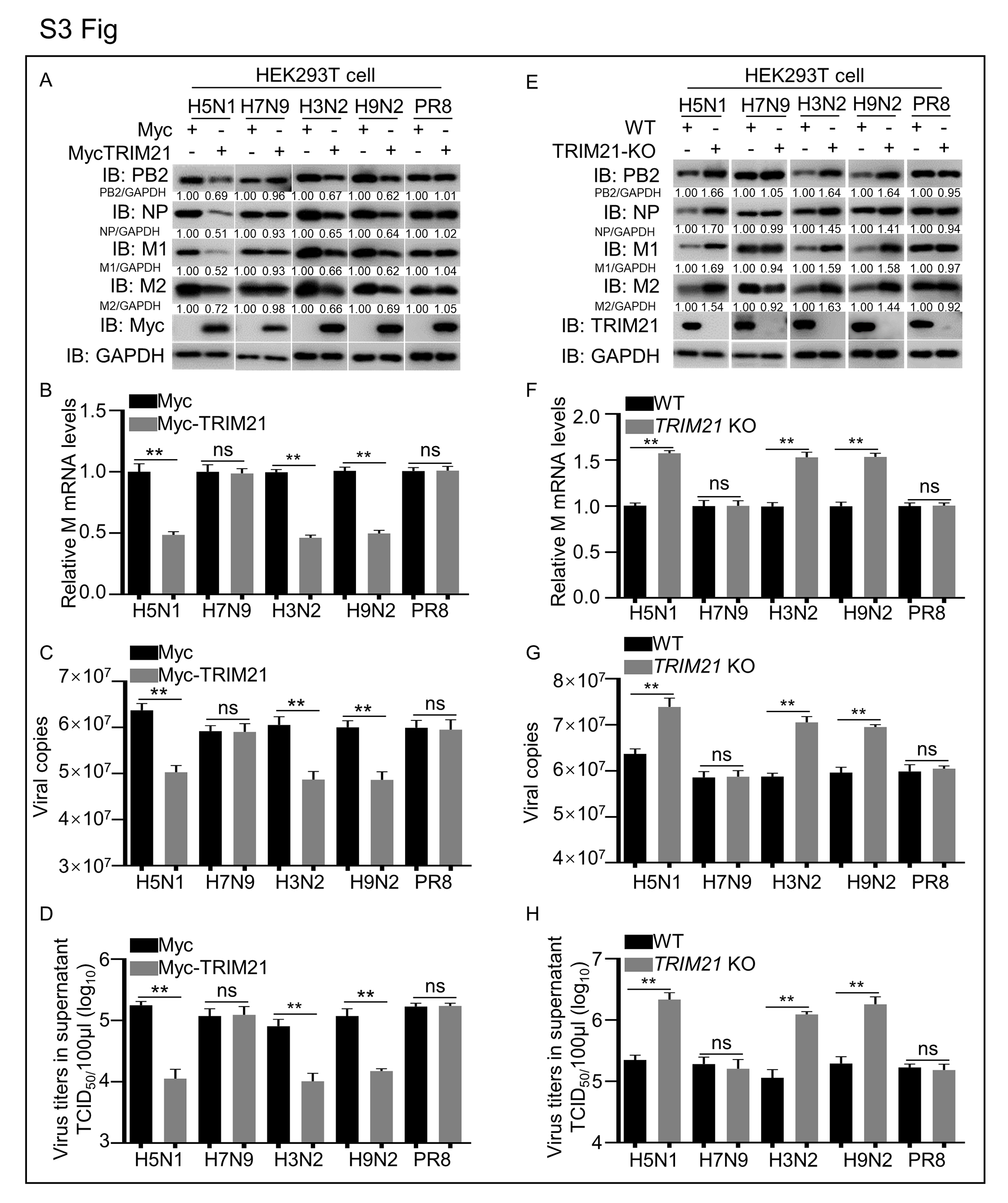

Supplement: S3 Fig — (A-D) TRIM21 inhibits the replication of H3N2, H5N1 and H9N2 influenza viruses in HEK293T cells, but has no effect on H7N9 and PR8. HEK293T cells were transfected with Myc-tagged vector or Myc-tagged TRIM21 for 24 h, the cells were then infected with H5N1, H7N9, H3N2, H9N2, PR8 at an MOI = 1.0 for 12 h, and the levels of viral proteins (A), M1 mRNA (B), vRNA (C), and the TCID50 (D) were examined. (E-H) TRIM21 increases the replication of H3N2, H5N1 and H9N2 influenza viruses in TRIM21-KO HEK293T cell lines. TRIM21-KO HEK293T cell lines were infected with H5N1, H7N9, H3N2, H9N2, and PR8 at an MOI = 1.0 for 12 h, and the levels of protein (E), M1 mRNA (F), vRNA (G), and TCID50 (H) were examined. Each experiment was independently performed with three biological repeats. All results are presented as means ± SD.*, p < 0.05; **, p < 0.01; ns, p > 0.05. (TIF) [file ppat.1011472.s003.tif]

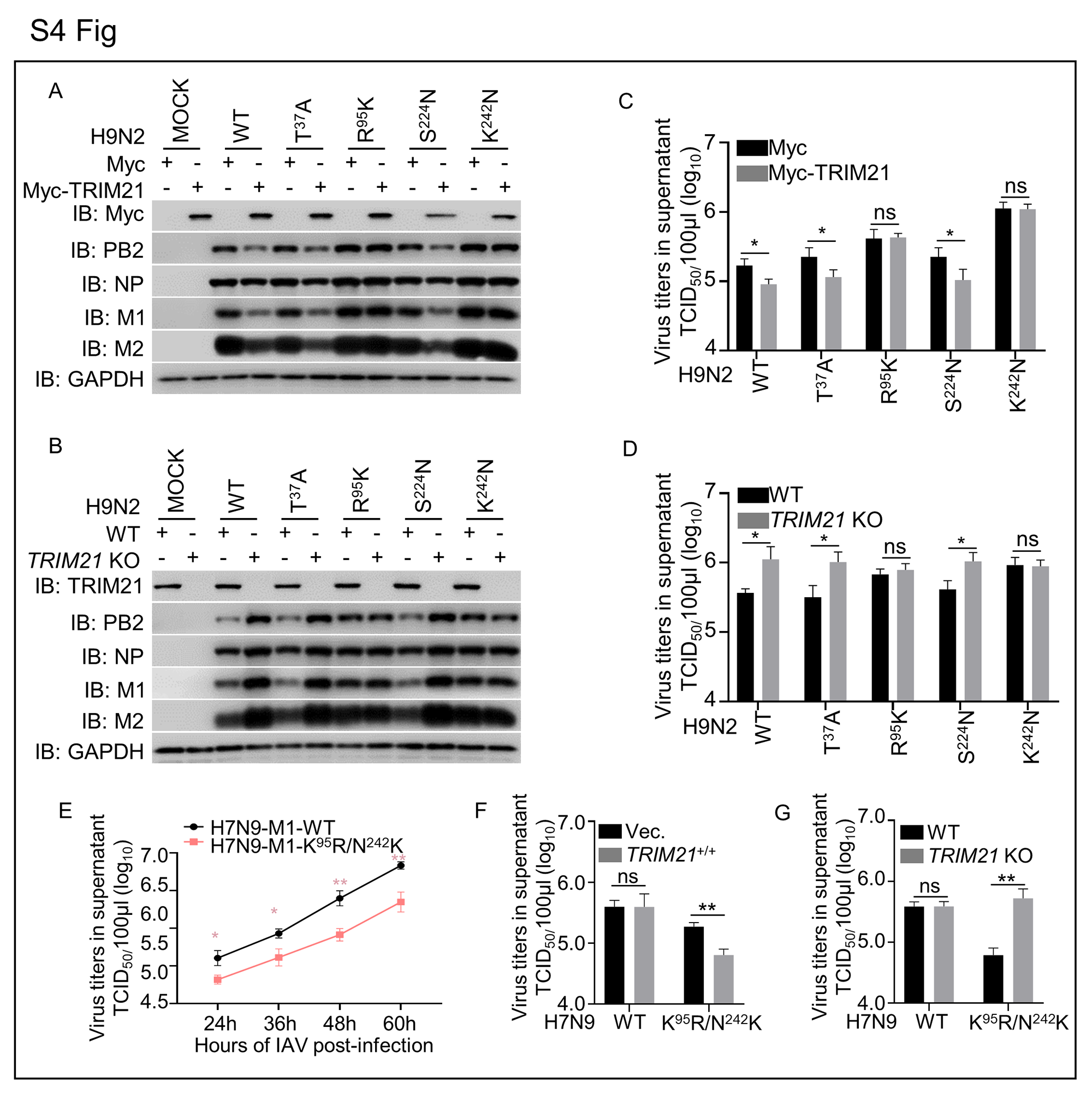

Supplement: S4 Fig — (A-D) TRIM21 restricted the replication of the WT and mutant viruses, except for the R95K and K242N viruses, in HEK293T cells. (A and C) HEK293T cells were transfected with Myc-tagged vector or Myc-tagged TRIM21 for 24 h, the cells were infected with WT H9N2 and mutant H9N2 (T37A, R95K, S224N, K242N) at an MOI = 1.0 for 12 h, and then the levels of viral proteins and viral titer were detected using western blotting and TCID50, respectively. (B and D) TRIM21-KO HEK293T cell lines were infected with WT H9N2 and mutant H9N2 (T37A, R95K, S224N, K242N) at a MOI = 1.0 for 12 h, and then the levels of proteins and viral titer were detected using western blotting and TCID50, respectively. (E) One-step growth curve of WT (H7N9-M1-WT) and mutant H7N9 virus (H7N9-M1-K95R/N242K). A549 cells were infected with H7N9-M1-WT and mutant H7N9-M1-K95R/N242K at an MOI = 0.01 for 24 h, 36 h, 48 h and 60 h, respectively, and TCID50 was measured as described in the Materials and Methods. (F-G) TRIM21 has no difference on H7N9-M1-WT replication in TRIM21-overexpressing and KO A549 cell lines. TRIM21-overexpressing, TRIM21-KO, and control A549 cells were infected with H7N9-M1-WT and H7N9-M1-K95R/N242K mutant viruses at an MOI = 1.0 for 12 h, and then the viral titer was detected using TCID50. Each experiment was independently performed with three biological repeats. All results are presented as means ± SD. *, p < 0.05; **, p < 0.01; ns, p > 0.05. (TIF) [file ppat.1011472.s004.tif]

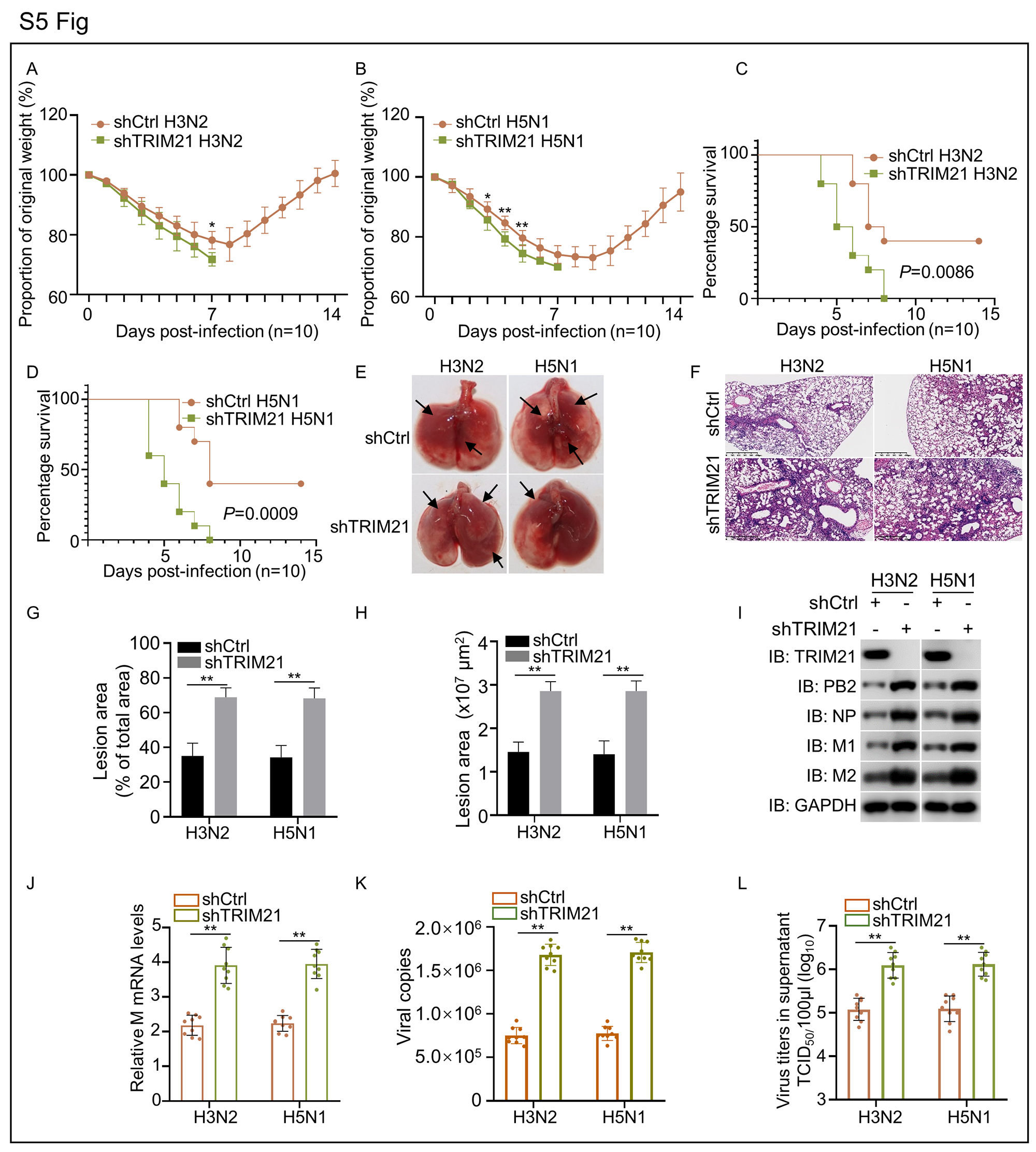

Supplement: S5 Fig — The 3-week-old C57BL/6 mice (thirteen per group) were intranasally infected with 1011.0 TCID50 of AAV6 with the TRIM21-shRNA (shTRIM21) or scrambled shRNA control (shCtrl). At four weeks after infection, the treated mice were intranasally infected with H3N2 (106.5 TCID50) and H5N1 (106.7TCID50). On day 6 post-infection, three mice per group were euthanized to check for lung lesions and virus replication, while the remaining ten mice per group were monitored until day 14. Mice with a weight loss exceeding 30% of their initial body weight were euthanized and recorded as dead. (A-D) Curves of body weight (A-B) and survival (C-D) in mice (mean ± SD; n = 10 mice) from day 0 to day 14 post-infection. (E-F) Gross (E) and histopathological (F) lesions in the lungs on day 6 post-infection. H&E staining was performed on lung sections (mean ± SD; n = 3 mice). (G-H) The lesion area was measured as a percentage and μm2 of the total lung area in (E-F). (I-L) The lungs were harvested to detect the levels of viral proteins (I), mRNA (J), vRNA (K), and the TCID50 (L) (mean ± SD; n = 3 mice). Each experiment was independently performed with three biological repeats. All results are presented as means ± SD. *, p < 0.05; **, p < 0.01; ns, p > 0.05. The photo by Lulu Lin. (TIF) [file ppat.1011472.s005.tif]

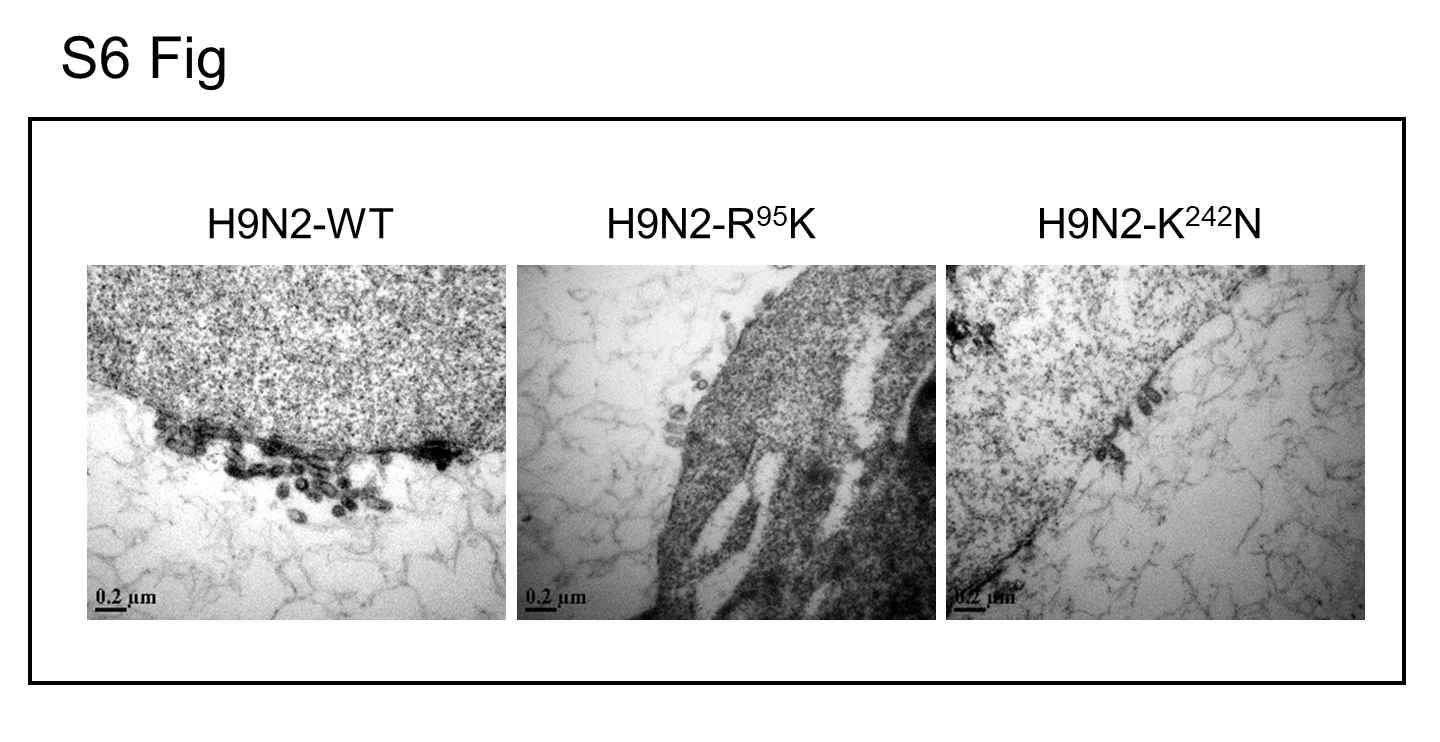

Supplement: S6 Fig — TRIM21-KO HEK293T cell lines were infected with H9N2-WT virus and mutant viruses (H9N2-R95K and H9N2-K242N) at an MOI of 20 for 10 h, and the cells were analyzed using transmissible electron microscopy. (TIF) [file ppat.1011472.s006.tif]
