# Supplementary material for: TRIM21 restricts influenza A virus replication by ubiquitination-dependent degradation of M1
Source: PLoS Pathog. 2023 Jun 21;19(6):e1011472. doi: 10.1371/journal.ppat.1011472 (PMC10325077; doi:10.1371/journal.ppat.1011472)
Supplement: S1 Table — (DOCX) [file ppat.1011472.s007.docx]

**S1 Table.** The primers used for cloning and quantitative real-time PCR.

| Name | Forward sequence (5’–3’) | Reverse sequence (5’–3’) |
| --- | --- | --- |
| TRIM21 | ATGGCTTCAGCAGCACGCTT | TCAATAGTCAGTGGATCCTT |
| TRIM21 △RING | TGGGAGGAGGTCACACGGCAGCGCTTTCTG | CAGAAAGCGCTGCCGTGTGACCTCCTCCCA |
| TRIM21 △BB | CACACAGGGGGAACGGGCCATGGTCCCTCTTG | CAAGAGGGACCATGGCCCGTTCCCCCTGTGTG |
| TRIM21 △CC | CACCGTGACCACGCCTGCCACAGCTCAGCAC | GTGCTGAGCTGTGGCAGGCGTGGTCACGGTG |
| TRIM21 △PRY/SPRY | GATATTACCTCTCCAAATATTGGATCACAAG | CTTGTGATCCAATATTTGGAGAGGTAATATC |
| H9N2 M1 | ATGAGTCTTCTAACCGAGGTCG | TCACTTGAATCGCTGTATTTGC |
| H9N2 M1 T^37^A | GTTTTTGCAGGGAAGAACGCCGATCTTGAGGCACTC | GAGTGCCTCAAGATCGGCGTTCTTCCCTGCAAAAAC |
| H9N2 M1 R^95^K | CAAACAACATGGACAAAGCAGTCAAGCTGTAC | GTACAGCTTGACTGCTTTGTCCATGTTGTTTG |
| H9N2 M1 S^224^N | GTCGGGACTCATCCTAACTCCAGTGCTGGTCT | AGACCAGCACTGGAGTTAGGATGAGTCCCGAC |
| H9N2 M1 K^242^N | GCAGGCCTACCAGAACCGGATGGGAGTGCAAA | TTTGCACTCCCATCCGGTTCTGGTAGGCCTGC |
| H9N2 M1 K^21^R | CGTCAGGCCCCCTCAGAGCCGAGATCGCGCAG | CTGCGCGATCTCGGCTCTGAGGGGGCCTGACG |
| H9N2 M1 K^35^R | GATGTCTTTGCAGGGAGGAACACCGATCTTGAG | CTCAAGATCGGTGTTCCTCCCTGCAAAGACATC |
| H9N2 M1 K^47^R | CTCATGGAATGGCTAAGGACAAGACCAATCCTG | CAGGATTGGTCTTGTCCTTAGCCATTCCATGAG |
| H9N2 M1 K^57^R | CTGTCACCTCTGACTAGGGGGATTTTAGGATTTG | CAAATCCTAAAATCCCCCTAGTCAGAGGTGACAG |
| H9N2 M1 K^98^R | TGGACAGAGCAGTCAGGCTGTACAGAAAGCTT | AAGCTTTCTGTACAGCCTGACTGCTCTGTCCA |
| H9N2 M1 K^101^R | CAGTCAAGCTGTACAGGAAGCTTAAAAGGGAAATA | TATTTCCCTTTTAAGCTTCCTGTACAGCTTGACTG |
| H9N2 M1 K^102^R | GTCAAGCTGTACAAGAGGCTTAAAAGGGAAATA | TATTTCCCTTTTAAGCCTTCTGTACAGCTTGAC |
| H9N2 M1 K^104^R | TGTACAGAAAGCTTAGAAGGGAAATAACATTCCATGG | CCATGGAATGTTATTTCCCTTCTAAGCTTTCTGTACA |
| H9N2 M1 K^113^R | ACATTCCATGGGGCAGAAGAGGTAGCACTCAG | CTGAGTGCTACCTCTTCTGCCCCATGGAATGT |
| H9N2 M1 K^187^R | GCCAGCACTACGGCTAGAGCTATGGAGCAGATG | CATCTGCTCCATAGCCCTAGCCGTAGTGCTGGC |
| H9N2 M1 K^230^R | CCAGTGCTGGTCTAAGAGATGATCTTCTTGAAAATTTG | CAAATTTTCAAGAAGATCATCTCTTAGACCAGCACTGG |

| H9N2 M1 K^242^R | GCAGGCCTACCAGAGACGGATGGGAGTGCAAA | TTTGCACTCCCATCCGTCTCTGGTAGGCCTGC |
| --- | --- | --- |
| H9N2 M1 K^252^R | CAAATACAGCGATTCAGGTGAGGTACCGCGGC | GCCGCGGTACCTCACCTGAATCGCTGTATTTG |
| PR8 M1 | ATGAGTCTTCTAACCGAGG | TTACTTGAACCGTTGCATC |
| H7N9 M1 | ATGAGTCTTCTAACCGAG | TCACTTGAACCGCTGCAG |
| H7N9 M1 K^95^R | CAAACAACATGGACAGGGCGGTTAAATTATAC | GTATAATTTAACCGCCCTGTCCATGTTGTTTG |
| H7N9 M1 N^242^K | GCAGGCCTACCAGAAACGGATGGGAGTGCAAC | GTTGCACTCCCATCCGTTTCTGGTAGGCCTGC |
| H3N2 M1 | ATGAGTCTTCTAACCGAGGTCG | TCACTTGAATCGTTGCATCTGC |
| H3N2 M1 T^37^A | GTCTTTGCTGGGAAGAACGCAGATCTCGAGGCTCTC | GAGAGCCTCGAGATCTGCGTTCTTCCCAGCAAAGAC |
| H3N2 M1 R^95^K | CAAATAACATGGACAAAGCAGTTAAACTGTAT | ATACAGTTTAACTGCTTTGTCCATGTTATTTG |
| H3N2 M1 S^224^N | ATTGGGACTCATCCTAACTCCAGTGCTGGTCT | AGACCAGCACTGGAGTTAGGATGAGTCCCAAT |
| H3N2 M1 K^242^N | GCAGGCCTATCAGAACCGAATGGGGGTGCAGA | TCTGCACCCCCATTCGGTTCTGATAGGCCTGC |
| H5N1 M1 | ATGAGTCTTTTAACCGAGGT | TCATTTGAATCGCTGCAGTT |
| H5N1 M1 T^37^A | GTTTTTGCAGGGAAGAACGCAGATCTTGAGGCTCTC | GAGAGCCTCAAGATCTGCGTTCTTCCCTGCAAAAAC |
| H5N1 M1 R^95^K | CAAACAACATGGACAAGGCAGTTAAATTATAC | GTATAATTTAACTGCCTTGTCCATGTTGTTTG |
| H5N1 M1 S^224^N | GTCGGGACTCACCCTAACTCCAGTACAGGTCT | AGACCTGTACTGGAGTTAGGGTGAGTCCCGAC |
| H5N1 M1 K^242^N | GCAGGCTTACCAGAACCGGATGGGAGTGCAAC | GTTGCACTCCCATCCGGTTCTGGTAAGCCTGC |
| *M1* mRNA | TTCTAACCGAGGTCGAAAC | CGTCTACGCTGCAGTCC |
| H9N2 WT virus *M1* mRNA | ATGGATAAAGACAAGACCAATCC | AGCTTCTTGTATAATTTAACTGAGC |
| H9N2 R^95^K virus *M1* mRNA | AGACCCAAACAACATGGAGTA | CCTTCTGCGGTCACTGTTCC |
| Human GAPDH | CATGAGAAGTATGACAACAGCCT | AGTCCTTCCACGATACCAAAGT |
| Mouse GAPDH | GTCAAGGCCGAGAATGGGAA | CTCGTGGTTCACACCCATCA |
